# Digital Health Interventions to Support Chronic Disease Management: Systematic Scoping Review

**DOI:** 10.2196/63742

**Published:** 2026-01-14

**Authors:** Abdullah Al Mahmud, Shane Joachim, Prem Prakash Jayaraman, Caitlin Learmonth, Shivani Tyagi, Abdur Rahim Mohammad Forkan, Muhammad Shuakat, Nilmini Wickramasinghe, Jack Wheeler, Stephanie Best, Alison Trainer

**Affiliations:** 1 Centre for Design Innovation, Department of Architectural and Industrial Design, Swinburne University of Technology, John St., Hawthorn, Melbourne, 3122, Australia, 61392143830; 2 Department of Computing Technologies, Swinburne University of Technology, Melbourne, Australia; 3 School of Science, Computing and Engineering Technologies, Factory of the Future and Digital Innovation Lab, Swinburne University of Technology, Melbourne, Australia; 4 Swinburne University of Technology, Melbourne, Australia; 5 Department of Communication Design, School of Design and Architecture, Swinburne University of Technology, Melbourne, Australia; 6 La Trobe University, Melbourne, Australia; 7 School of Computing, Engineering & Mathematical Sciences, La Trobe University, Melbourne, Australia; 8 Parkville Familial Cancer Centre, Peter MacCallum Cancer Centre, Melbourne, Australia; 9 School of Health Sciences, University of Melbourne, Melbourne, Australia

**Keywords:** mHealth, chronic disease, digital technology, co-design, user-centered design, user centered, chronic disease management, support, scoping review, design, development, digital health platform, qualitative content analysis, web-based, self-management, quality of life, clinical utility, patient communication

## Abstract

**Background:**

Health interventions delivered by digital platforms are gaining popularity and are evolving to address the needs of patients with chronic diseases. The heterogeneity of chronic diseases requires that digital health platforms vary in their approaches to chronic disease management.

**Objective:**

This review aimed to explore the characteristics of digital health platforms and the corresponding digital interventions developed to support patients with chronic diseases. This includes those platforms’ design, development, and the metrics by which any incremental benefits they provide are assessed.

**Methods:**

We searched electronic databases including Scopus, Web of Science, PsycINFO, IEEE Xplore, MEDLINE, and Embase. Relevant articles published from January 2013 to November 2024 were extracted. Extracted data were then synthesized using qualitative content analysis and presented in narrative form with relevant tables.

**Results:**

In total, we identified 69 digital health platforms supporting the management of 20 chronic diseases. Most platforms were mobile apps (n=22) or a combination of web and mobile apps (n=15). Most of the platforms (n=44) were tailored to support self-management of chronic diseases. These platforms also provided a web-based portal where health care providers could review and manage the information recorded by patients. In 77% (53/69) of the studies, patients reported that the digital interventions delivered by the platform improved their quality of life, their health, and their ability to self-manage their chronic diseases. In addition, health care providers reported positive outcomes, including improved clinical utility and patient communication. While short-term health outcomes of the digital health interventions were largely positive, long-term health outcomes remain unknown. This was because most of the studies were short-term pilots and often formative in nature (n=42). Many had limited sample sizes, limited participant uptake of the digital platforms, and technical issues. In many cases, further personalization of platforms was required to meet patients’ self-management needs.

**Conclusions:**

Digital health interventions can be beneficial in the management of chronic disease. The adoption of digital interventions in combination with regular clinical care can improve health outcomes, support self-management, and enhance communication between patients and health care providers. However, long-term user engagement is the major barrier to their long-term success. High dropout rates, often resulting from a lack of motivation or technical issues, testify to the need for adaptive, low-burden interventions that function seamlessly in users’ daily lives. Adopting user-centered and co-design approaches that engage both clinicians and patients in designing digital health platforms may enhance the usability and uptake of such platforms.

## Introduction

### Background

Chronic diseases are defined as long-lasting conditions (ie, 1 y or more) and require ongoing care and account for 41 million (74%) deaths worldwide [1-3]. They can cause disability that affects the quality of life and reduces life expectancy. Patients with chronic diseases may face several challenges when managing their condition, such as (1) conflicting knowledge about the disease or how to manage it, (2) access to care, and (3) communication with health care providers [4]. Chronic diseases pose a significant burden on health care systems, families, and caregivers [5]. Therefore, the prevention and management of chronic diseases has become a global priority, as the prevalence of those diseases can undermine social and economic development.

Digital health interventions use technology to deliver health care services or treatments and facilitate knowledge exchange [6]. These interventions are designed to enhance the quality of patient care by capturing and conveying information in a digital format. Digital health interventions may involve electronic medical records (EMRs), mobile apps or web applications, and wearable sensors such as Fitbit (Google). Technologies such as digital health platforms are gaining increased use in managing chronic diseases [7]. The proliferation of mobile apps and the ubiquitous nature of information technology have fueled the development of platforms that support the management of chronic disease [8,9]. The digital health technologies discussed in this study include mobile apps, web applications, electronic health records (EHRs), EMRs, wearable devices, and telehealth services [10]. We have used digital platforms as an umbrella term throughout the paper to denote these technologies.

With the rapid growth of digital platforms for chronic disease management, a systematic synthesis of these platforms is needed to inform effective and efficient care. Recent studies have investigated the framework for managing chronic diseases [11] and the potential of technology adoption [12]. To our knowledge, no studies have examined how digital platforms support the management of chronic diseases. Due to the heterogeneity of digital platforms, it is vital to investigate the types of digital platforms that are available and the usability and acceptability of these platforms. In addition, it is important to investigate the processes that led to their design and development, as well as the metrics used to assess their benefits. In this study, digital platforms are characterized as assortments of web-based and mobile applications and related technologies that are used to deliver health care services [13].

### Aims

This review aims to explore the characteristics of digital health platforms and corresponding digital health interventions that support patients with chronic disease. This exploration will include those technologies’ design, their development processes, and the metrics by which their incremental and long-term benefits have been assessed.

## Methods

### Overview

This review adopts the scoping review methodology proposed by Arksey and O’Malley [14] because we are interested in identifying and mapping emerging evidence [15]. PRISMA-ScR (Preferred Reporting Items for Systematic reviews and Meta-Analyses extension for Scoping Reviews) checklist [16] was used throughout the review to ensure adherence (Checklist 1). However, there are some differences between the registered protocol and this paper regarding database searching. Furthermore, 2 additional databases (Medline and Embase) have been searched, and the search period has been extended to November 2024 for all the databases.

### Step 1: Identifying the Research Questions

First, what are the characteristics of digital platforms that support the management of chronic diseases, including self-management and provider-led management?

Second, what principles and theoretical frameworks have been used to design or co-design these platforms?

Third, how were these platforms evaluated for clinical utility?

Finally, what is the effectiveness of those platforms?

### Step 2: Search Strategy

The search terms used for the literature search are “chronic disease” OR “chronic illness,” OR “long-term conditions,” OR “chronic conditions” AND “Digital” OR “mHealth” OR “App” AND “management.”

We did not include the exact term “self-management” in our search, nor the standard indexing terms that databases use for that idea (eg, PubMed’s MeSH heading Self Care and Embase’s Emtree term self-management). To reduce the chance of missing papers, we implemented citation chasing, that is, for each included study, we checked its reference list and looked up newer papers that cite it.

Initially, databases such as Scopus, Web of Science, PsycINFO, and IEEE Xplore were searched from January 2013 to 30 November 2022. Later, an additional search was conducted on these databases from November 2022 to November 2024. Furthermore, 2 additional databases, MEDLINE and Embase, were searched from January 2013 to November 2024 (Refer to Multimedia Appendix 1 for the search outcomes). Articles retrieved were imported into Covidence software (Veritas Health Innovation Ltd) [17], and duplicated items were automatically removed.

### Step 3: Study Selection

The following inclusion and exclusion criteria were used.

#### Inclusion criteria

First, the study must be a peer-reviewed journal article and present primary data. Second, it should be published within the last 12 years (January 2013 to November 2024). We chose 2013 as the starting year to reflect a critical turning point in the evolution of digital health technologies. Around this time, the widespread use of smartphones and mobile apps, breakthroughs in wearable sensors, and more prevalent usage of EHRs began to transform chronic disease management [18,19]. Third, it should be available in English. Finally, it should involve digital applications in the context of chronic disease management.

#### Exclusion criteria

The study was excluded if (1) it was a review article or opinion piece, or (2) hypothetical use of digital technology was found in it.

Initially, titles and abstracts were reviewed against the selection criteria and were marked as “include,” “exclude,” or “uncertain.” Two reviewers (AAM and MS) conducted the screening independently, and regular discussions with the research team were undertaken to resolve any discrepancies and to fine-tune selection criteria. This screening and discussion process continued until we reached a consensus. Subsequently, for the included studies, a full-text review was carried out (AAM and MS) against the selection criteria following the same screening procedure.

### Step 4: Data Extraction and Charting the Data

Two authors (AAM and MS) developed a data charting form to identify relevant information to extract from the included studies. Using this form, the following data were extracted: study citation, publication type, authors, study location, study year, user acceptability of the digital interventions or digital platforms, and outcome of the study (quantitative results, qualitative themes, recommendations, key learnings, and limitations). AAM and MS charted the extracted information.

### Step 5: Collating, Summarizing, and Reporting the Results

First, the extracted data were analyzed using descriptive statistics (eg, frequencies). This provided numerical summaries of (1) digital platforms and their characteristics, (2) chronic diseases, (3) platform design principles, and (4) outcomes. These details were presented using tables, charts, and graphs, followed by a summary. Second, 2 authors (AAM and CL) independently analyzed the extracted data thematically to identify themes. Results from the 2 reviewers’ thematic analysis were combined to select the final collection of themes.

## Results

### Overview

**Figure 1.**
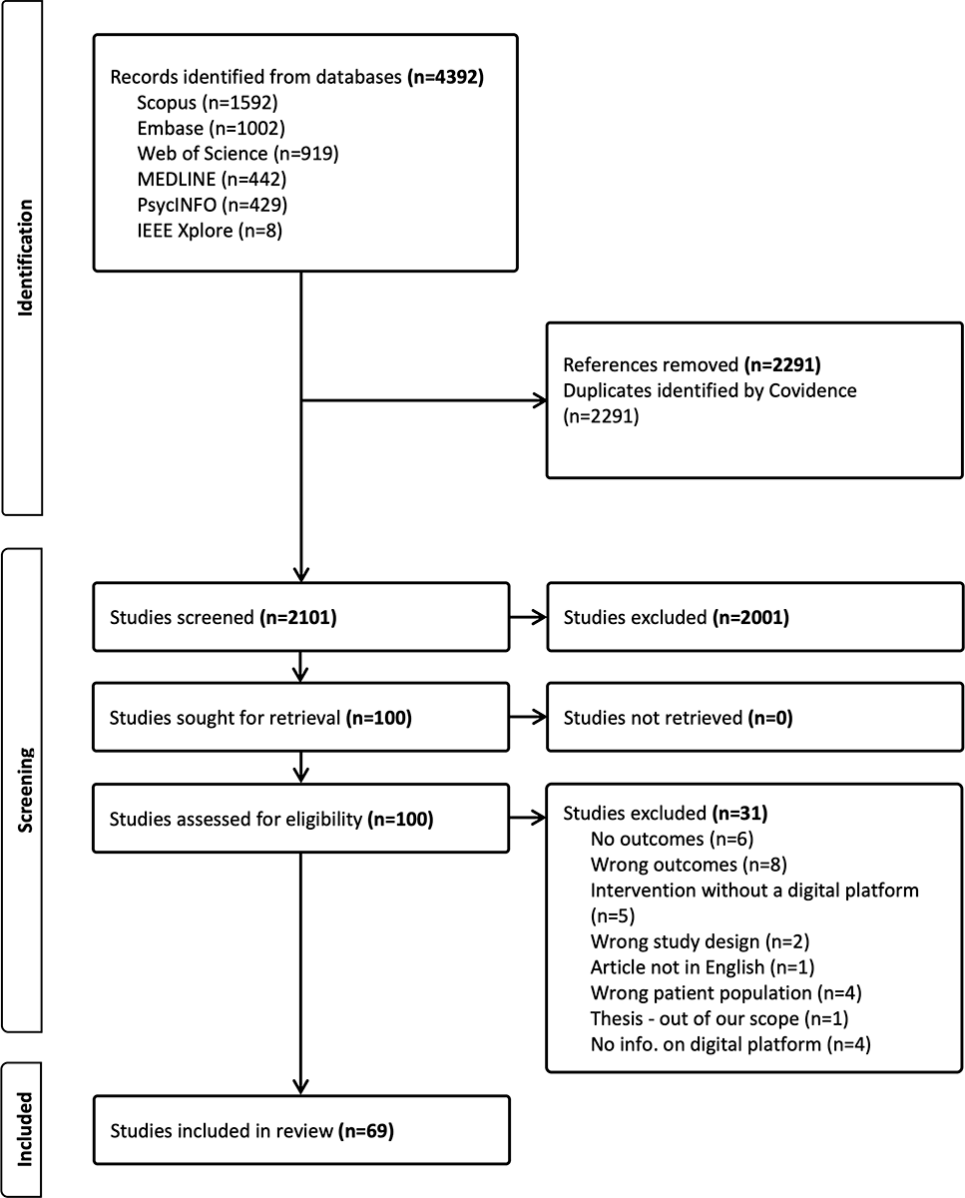
PRISMA diagram showing the article selection process.

A total of 4392 studies were identified from the 4 databases. Of these, 2291 were duplicates, leaving 2101 to be screened. In total, 2001 studies were excluded during the title and abstract screening process, and 100 were assessed for eligibility. Of these, 69 studies [20-88] met our eligibility criteria and were included for review. Refer to Figure 1 for the article selection process.

### Characteristics of the Included Studies

In total, 69 studies were conducted in 26 countries. Of the total, 15 studies (22%) were conducted in the United States [22,25-27,30,31,37,38,49,50,52,55,61,66,85]. Canada had 5 studies (7%) [38,49,50,76,84], and China had 10 studies (15%) [24,32,41,43,58,63,65,69,79,81], while 4 studies (5%) [28,59,67,83] were conducted in Spain. The most common chronic disease reported was type 2 diabetes (n=17, 20%), followed by heart failure (n=11, 13%), chronic obstructive pulmonary disease (COPD; n=9, 11%), and hypertension (n=9, 11%) (Multimedia Appendix 2). In total, 27 studies (39%) [21,22,24,27-29,31,33,34,37,41,42,45,49,50,52,54,55,59,60,63-69] are reported as randomized controlled trials (RCTs), and the remaining 42 studies (61%) are formative studies [20,23,25,26,30,32,35,36,38-40,43,44,46-48,51,53,56-58,61,62,70-88].

Table 1 summarizes the aims of the digital platforms, the platform types, and the study settings.

**Table 1.** Summary of the platforms.

Platform characteristics	Total, n (%)
Aim of the digital platform (n=77)	
Self-management^a^	44 (57)
Behavior changes	17 (22)
Communication with health care providers	16 (20)
Types of digital platforms (n=69)	
Web-based application	15 (22)
Mobile app	33 (48)
Combination of web-based application and mobile app (ie, multimodal)	14 (20)
Other^b^ (SMS text messaging)	7 (10)
Wearable device (in combination with web app, smartphone app, or both)	16 (23)
Study setting (n=78)	
Hospital or primary care setting	34 (44)
Home	36 (46)
Online Community	8 (10)

a Includes self-monitoring of symptoms, medication, physical activity, etc.

b One intervention used telehealth and SMS text messaging.

### Characteristics of the Study Participants

All the included studies had adult participants (aged >18 y). Participants from various age groups were included in the studies (30‐35 y: n=2, 40‐45 y: n=1, 45‐50 y: n=1, 60 y or older: n=19, and 75‐80 y: n=1). Several studies (n=15) did not specify the mean age or age range. Most of the studies are dominated by female participants, especially studies related to the management of chronic diseases such as asthma (eg, 80 women vs 26 men [20]), hypertension (eg, 49/67, 73% female [21]), diabetes, and rheumatoid arthritis. Some studies target specific gender groups to address health disparities or disease prevalence, such as Black women with hypertension [22]. Most chronic conditions are higher in females, which may be a reason for overrepresentation. Some studies, like those dealing with heart failure (eg, N=25, 100% male participants [23]), atrial fibrillation (eg, 59/96, 61.5% male [24]), and other cardiovascular disorders (eg, 48/79, 61% male [54]), might have a greater proportion of males. In some instances, these conditions may be more prevalent in men within specific age groups. Most male-dominated studies occur in areas such as cardiovascular health, where men are more prone to certain conditions.

In total, 6 studies [40,54,57,58,71,89] mentioned the literacy levels of participants in the participant eligibility criteria. Only 1 study [26] mentioned digital literacy (ie, technical and cognitive abilities to use information and communication technologies [89]), defined as “acceptable literacy level to read and write with a smartphone,” in the inclusion criteria. None of the studies measured the participants’ digital literacy level as part of the intervention. Most studies (n=19; 61%) had predominantly male participants (66%). Where literacies were mentioned, the most reported literacy was linguistic (English language literacy, n=2 out of 6), followed by health literacy [90], defined as the capability to process and understand health information (n=2 out of 6).

### Characteristics of the Digital Interventions

The digital interventions identified in the literature targeted a range of chronic diseases and thus had a variety of operational aims. Multimedia Appendix 3 provides a summary of the digital intervention strategies used in the studies. The definition of each digital intervention is extracted from the National Institute for Health and Care Excellence (NICE) Framework [91]. An analysis of the digital intervention strategies based on the NICE Evidence Standards Framework for digital health [1] found self-management to be the most prevalent strategy, appearing in 68 studies (99%) [20-46,49-86,88]. Collaborative care (ie, self-management plus provider-led management) followed with 43 occurrences (39%) [20-25,27,28,32-37,39-42,44,51-53,56,57,60-63,65,67,69,70,72,74,77-83,85,87], while information and education were present in 27 studies (39%) [20-24,26,33,37,40,45,46,57,59,63,64,66,72-79,81,84,85]. Personal health record systems were identified in 25 papers (62%), digital therapeutics in 15 papers (22%) [22,23,25,28,35,44,45,51,59,60,63,66,72,73,77], clinical decision support systems in 8 papers (12%) [26,28,44,48,59,65,70,72], and active monitoring in 1 paper (2%) [30].

Self-management frequently co-occurs with collaborative care (47 times), reflecting strong integration between patient-driven health management interventions and the need for clinical support (ie, provider-led management). Information and education (31 times) and personal health record systems (25 times) also frequently co-occur with self-management, underscoring the importance of providing users with relevant knowledge and real-time data to make informed health decisions. This, in turn, enhances self-care practices and promotes better health outcomes. Additionally, digital therapeutics (15 times) and clinical decision support systems (8 times) demonstrate strong associations, emphasizing their role in remote treatment guidance and data-driven clinical decision-making. These findings highlight how personalized digital interventions, combined with real-time insights, can enhance both patient engagement and clinical oversight.

### Features of the Digital Platforms and Behavior Change Techniques Offered

#### Overview

The most common features by far are self-monitoring and tracking (65/69 studies, 94%), showing the trend of users’ empowerment for active management (Table 2) of chronic diseases with support from health care providers. Medication reminders (59/69 studies, 86%) and behavioral support features (48/69 sources, 70%) are essential in helping patients stay committed to their treatment plans, with alerts (59/69 studies, 86%) and gamification using points, badges, or levels (8/69 studies, 12%) playing key roles in engagement. Most of the platforms are also targeted at improving user awareness and engagement by educating the users about better health practices (55/69 sources, 80%) and communicating with health care providers (45/69 studies, 72%). Such features ensure that patients are supported not only through technology but also by and through human contact.

**Table 2.** Summary of the most common features across the 69 studies, including their frequencies.

Category and feature	Frequency
Mobile app (n=32)	
Self-monitoring	15
Patient education	10
Reminders	18
Data tracking	14
Communication (between patient and health care providers)	12
Personalized feedback	8
Medication adherence	7
Activity tracking	9
Health reporting (eg, symptom, weight, and blood pressure)	6
Push notifications	7
Web-based application (n=15)	
Health data management	8
Patient education	5
Health reporting	6
Care team communication	4
Goal setting	3
Progress tracking	4
Data visualization	2
Document management (eg, laboratory results and prescriptions)	2
Personalized feedback	1
Combination of web-based application and mobile app (multimodal; n=16)	
Health data integration	7
Communication (between patient and health care providers)	5
Health tracking	6
Goal setting	5
Personalized feedback	4
Reminders and notifications	5
Symptom tracking	4
Educational content	3
Activity tracking	3
Wearable device (in combination with web application or mobile app; n=16)	
Physical activity tracking	9
Health data measurement (eg, heart rate, blood pressure, oxygen saturation, and SpO2^a^)	8
Real-time monitoring	7
Data syncing (with mobile app or cloud)	6
Reminder and alert functionality	5
Health reporting	4
Medication reminders	3
Data visualization	3
Other (SMS text messaging; n=7)	
Reminders (medication, appointments, etc)	7
Behavioral triggers	5
Education (health tips and guidance)	6
Symptom reporting	4
Daily check-ins or reports	3

a SpO2: peripheral oxygen saturation.

Table 2 provides a summary of the features and their frequencies by category. Routine input of health data was facilitated through surveys and questionnaires, using free-text or drop-down menu functionalities. Some apps facilitated image uploads, allowing patients to supply photos of wounds, rashes, or other relevant aspects of their conditions to be assessed by health care providers [27,28]. To support the self-management and tracking of health data, many platforms use tools for self-measurement and reporting of key health indicators, such as blood pressure (BP), glucose levels, and heart rate. Furthermore, 2 platforms embedded these functionalities in motion sensors [29,30]. However, the majority of platforms incorporating self-reporting functionalities did so through Bluetooth-enabled technology, such as smart watches, BP monitors, and scales [31-36]. These either fed data directly to the platforms or provided data for participants to input manually. Refer to Table 3 for the overview of the wearable devices used in the included studies.

**Table 3.** Wearable devices used in the included studies (n=16).

Wearable device	Type of wearable	Purpose	Study
Fitbit	Activity tracker or fitness band	General health monitoring, physical activity, and heart rate tracking	Oh et al [34]
Apple Watch	Smartwatch	ECG^a^, heart rate monitoring, activity tracking, and general health monitoring	Guo et al [32]
Omron BP Cuff	Blood pressure monitor	Blood pressure tracking and monitoring for hypertension	Evans et al [30]
Dexcom CGM	Continuous glucose monitor	Continuous glucose monitoring for diabetes	Schnall et al [37]
iHealth Pulse Oximeter	Pulse oximeter	Monitoring oxygen levels (SpO2^b^) for respiratory conditions	Guo et al [32]
AliveCor KardiaMobile	ECG monitor	ECG readings for detecting arrhythmias	Gray et al [38]
Masimo Pulse Oximeter	Pulse oximeter	Monitoring oxygen saturation levels (SpO2)	Kryger et al [27]
Apple Watch	Smartwatch	Heart rate monitoring, ECG, and wellness tracking	Burda et al [39]
Fitbit	Activity tracker or fitness band	Step counting, heart rate monitoring, and sleep tracking	Bailey et al [40]
FreeStyle Libre	Continuous glucose monitor	Continuous glucose monitoring for diabetes management	Dorsch et al [31]
Google Glass	Smart glasses	Augmented reality for hands-free monitoring during medical procedures	Zhang et al [41]
Quell	Pain management wearable	Nerve stimulation for chronic pain relief	Cormican and Dowling [88]
Oura Ring	Wearable sleep tracker	Sleep quality tracking and recovery monitoring	Poppe et al [42]
Masimo	Pulse oximeter	Continuous SpO2 monitoring for respiratory health	Ji et al [43]
WHOOP Strap	Wearable fitness tracker	Sleep and recovery tracking for physical performance enhancement	Doyle et al [44]
iHealth Pulse Oximeter	Pulse oximeter	Monitoring oxygen levels in the blood	Ji et al [43]

a ECG: electrocardiogram.

b SpO2: peripheral oxygen saturation.

Only 5 studies [29,44-47] explicitly mentioned the use of artificial intelligence (AI) in the digital platforms. In these, AI was used to aggregate and analyze patient data across multimodal platforms. This was achieved through machine learning algorithms or recommender systems on platforms that use conversation agents. For example, the Snapcare app (Snapcare Technologies Pvt Ltd) gathered daily activity data over 12 weeks to address chronic back pain, including walking distance and workouts [29]. Notifications were sent to patients based on app usage and physical activity data collected by built-in phone sensors. This information was automatically transferred to a secure server, where machine learning algorithms examined the daily data on physical activity and produced suggestions for the session the following day. Similarly, the ProACT digital platform helps older patients manage their multimorbidity, including diabetes, congestive heart failure, COPD, and chronic heart disease [44]. Health information, including BP, heart rate, blood glucose, pulse oximetry, weight, activity, and sleep readings, is collected using off-the-shelf technologies. ProACT’s AI algorithms can gain knowledge from the data to provide more accurate personalized recommendations and highlight a condition that needs attention. Likewise, using an embodied conversational agent named Laura, My Diabetes Coach (The University of Melbourne) provides patients with individualized support, monitoring, and motivational coaching [45]. The algorithms and conversational scripts that direct each person’s progress were developed using behavior change theories, and they can accommodate recommendations by a general practitioner. Natural language processing and automated speech recognition are used to enhance the capability of a voice-enabled chatbot in user interactions in French [46]. The Medly voice app (University Health Network) for heart failure management leverages various AI technologies, including machine learning, natural language processing, and automated speech recognition, which process the speech of users and formulate responses [47].

In total, 14 of the platforms included in the review were multimodal. For example, a platform might function as a combination of web-based and smartphone-based applications or work in combination with a wearable Bluetooth device, such as a smartwatch or BP monitor. Of the total, 8 interventions included some form of wearable technology or measurement device. These ranged from smartwatches and activity trackers [30-32,40,44] to BP monitors [33,34,36,44], glucose monitors [34,44], scales [31], and sleep monitoring devices [44]. Additionally, 15 interventions [26-28,35,37,39,44,47,48,50,53,58,61,62,71] were designed to work in conjunction with in-person consultations. These included functionalities that were integrated with routine clinical visits to monitor appointments, provide technical support, and review progress. Digital platforms in 10 studies supported multimorbidity [27,34,35,38,44,48-52].

Behavior change was broadly reported to be a multistep process requiring numerous complementary features. Tailoring features to the specific needs of participants based on both demographics and disease type was shown to be important in achieving behavior change [32,48,53]. Although not all of the studies explicitly used theoretical frameworks for behavioral change, many of the platforms are underpinned by theories intended to enable behavior change. Theories applied include social cognitive theory, which is detailed in the work of Dale et al [54], and the transtheoretical model, which is elaborated upon by Salari et al [53]. Furthermore, Sittig et al [55] explore the Fogg Behavior Model, and both cognitive theory and self-efficacy theory are examined by Park et al [60].

A range of methods was used to deliver tailored behavior change features to facilitate user engagement and improved health outcomes. Reminders, goal-setting, and motivational messages were widely used in 5 studies [38,39,44,56,88]. Those features aimed to motivate patients to engage with their health interventions and help them stay on track with their treatment plans. Behavioral trigger messaging was used to promote engagement, motivation, and self-belief. It was also used to provide reinforcement to participants based on behavior change theories [55]. Similarly, theory-based approaches specific to tailored SMS text messaging were used to improve personalization and increase the acceptability of the health interventions [32,53].

Gamification and learning material were incorporated in some studies for the optimization of motivation and to increase users’ knowledge of managing their conditions. Gamification, either through rewards or challenges, stimulated usage of the app, while educational information [26,33,40,57] provided valuable information to allow patients to better understand their conditions and make informed decisions on their treatment. Furthermore, 5 platforms used gamification techniques to enhance user experience while embedding learning principles [29,42,45,58,59]. In-app conversation agents supported by AI [45] were used in a platform to change behavior too.

Furthermore, self-monitoring, tailored feedback, and social support were key features of some platforms [26,35,40,60]. These features fostered engagement and behavior change. Self-monitoring permitted users to monitor their health measurements (ie, BP or blood glucose), while personalized feedback assisted in matching interventions to individual needs and encouraging ownership of one’s health. Social support, either through peer interaction or direct contact with health care providers, also facilitated long-term engagement by providing users with emotional encouragement and accountability.

#### Chronic Disease Management: Patient Self-Management and Provider-Led Care

In analyzing digital platform features, we distinguish 2 complementary approaches: patient self-management and provider-led management. Provider-led management refers to the coordinated, proactive activities delivered by health care services and clinicians to support people living with chronic conditions, typically including comprehensive care planning, risk stratification, guideline-concordant treatment, continuity, between-visit care coordination, and use of clinical information systems for monitoring and quality improvement across settings and providers [92]. In contrast, patient-led self-management encompasses the day-to-day work undertaken by people living with chronic conditions, including symptom monitoring, medication adherence, lifestyle and behavioral changes, decision-making, help-seeking, and the usage of tools and supports, including digital platforms [93].

The vast majority of digital platforms (n=36) were designed to be used at home by patients, and this was usually done in conjunction with their ongoing health care plans. To support at-home interventions, some platforms incorporated automated SMS text messaging, including conversational agents and trigger SMS text messaging [45,55]. The key focus of platforms that were exclusively home-based was self-management through input and self-monitoring of health data. Of the 36 interventions, 13 designed to be used in the home were also implemented in primary care settings, including hospitals. The digital platforms, which contained a patient-facing mobile health (mHealth) app, primarily focused on self-management of the symptoms of a chronic condition. The data collected by an mHealth app is usually passed to a practitioner-facing portal, where the practitioner can view patient data and adjust care plans. In some cases, these digital platforms supported bidirectional communication, allowing the practitioner to directly communicate with patients through the platform [32,35,45,53,60,61]. A few platforms also supported the booking of appointments with relevant health care providers [33,48,49] or sent reminders about upcoming appointments [43].

Because a high number of digital platforms are tailored to support self-management (n=44), the majority of platforms were primarily managed by the patients themselves. However, where the digital platforms were multimodal, consisting of a smartphone app and a web-based application, the smartphone apps tended to be managed by the patients, while the web-based applications were used by health care providers to review and manage patient information through an online portal.

#### Principles Used in the Design and Development of the Digital Platforms

In total, 25 studies (36%) used some form of co-design, consultative, or user-centered approach to developing the digital platforms. Furthermore, 11 studies focused on platforms that either had already existed or were adapted from existing models. Co-design approaches varied and included expert consultation with health care providers and user-centered iterative approaches, which sought feedback from patients throughout an intervention period.

Most of the platforms were developed by using surveys or focus groups to gather information and determine requirements. Often, these surveys and focus groups lacked the full participation of end users. In some studies, the iterative development approach also included gaining input from caregivers, patients, and related stakeholders. For example, the iMHere 2.0 [35] system was iteratively designed, developed, and evaluated with patients involved at all stages. Other studies that discussed the development of digital platforms did not follow or report on the iterative development process. One platform [62] was not accepted as it failed to meet the needs of the patients. Studies adopting an iterative approach tended to make changes to the digital intervention throughout the testing period in response to feedback, whereas those using professional consultations at the beginning and end of the process did not make changes during the testing period. Platforms primarily seeking to influence behavior change tended to be developed using more theoretically based approaches, such as behavior change models and social cognitive theory [54,55].

### Outcomes of Studies Assessing the Digital Platforms

Outcomes of the digital platform assessments reported in the studies are grouped into 2 categories: outcomes of randomized controlled studies and other formative studies.

### Outcomes of RCTs

The majority of the included studies demonstrated significant benefits and health outcomes, such as symptom reduction, better disease control, and improved quality of life. A smaller proportion of studies showed no significant effect on the primary outcomes, with improvements in only secondary measures that did not significantly impact the main health indicators. Some trials did not have the expected results, sometimes due to high dropout rates, lack of long-term effects, or problems with patient engagement (Table 4).

**Table 4.** Summary of the randomized controlled trial studies (n=27).

Study	Favorable outcomes	No effects	Name of the platform and target disease
Li et al [63]	Significant improvement in disease control at 6 months (*P*=.001).	At 12 months, no significant difference between groups (*P*=.90).	A smart system of disease management; rheumatoid arthritis
Goulding et al [64]	Decreased relapse risk in the low-risk group (*P*=.02), improved depressive symptoms (*P*=.02), and relational quality of life (*P*=.02).	There was no significant improvement in relapse risk for the high-risk group (*P*=.62).	A smartphone-based self-management intervention (LiveWell) app and website; bipolar disorder
Goodman and Locke [21]	Significant improvements in blood pressure were found, with systolic BP^a^ decreasing from 140 to 134 mm Hg (*P*=.001) and diastolic BP decreasing from 78 to 74 mm Hg (*P=*.007)	No effect on the usual care group for HbA1c^b^ (*P*=.19).	A mobile phone-delivered diabetes intervention; diabetes
Zhang et al [65]	Significant improvements in HbA1c (*P*<.05), diastolic BP (*P*<.05), and fasting plasma glucose (*P*<.05).	No effect on BMI.	A digital health technology to provide shared decision-making–informed dietary intervention; diabetes
Buis et al [66]	Both groups showed significant reductions in systolic BP (*P*<.001).	No significant differences between groups for BP or other outcomes (*P*=.99).	MI-BP, a culturally tailored multibehavior mobile health intervention; hypertension
Zhang et al [41]	Significant improvement in HbA1c (*P*<.001) and ABC^c^ control rate (*P*=.025). ABC goals are HbA1c <7%, systolic BP/diastolic BP <140/80 mm Hg, and LDL-C^d^ <2.6 mmol/L.	No changes in LDL-C or blood pressure (*P*=.95).	SMARTDiabetes app; diabetes
Tabernero et al [67]	Significant improvement in emotional well-being and self-efficacy for chronic disease management (*P*<.05).	No effect on anxiety and depression levels.	Psychological interventions delivered via mHealth^e^ technology; chronic cardiac diseases
Hartch et al [52]	Significant improvements in medication adherence (Cohen *d*=−0.52, *P*=.014) and medication self-efficacy (Cohen *d*=0.43, *P*=.035).	No significant effect on medication knowledge or social support (*P*=.15).	Medisafe app; hypertension, diabetes, and asthma
Xu et al [24]	Significant improvement in anticoagulation knowledge, medication compliance, and patient satisfaction (*P*<.05).	No effect on bleeding or thrombotic events.	Alfalfa app; atrial fibrillation
Abel et al [22]	Significant reduction in systolic BP (*P*<.001), weight (*P*<.001), and physical activity (*P*=.018).	No significant difference in BP control between the treatment and control groups (*P*=.99). No sustained effect on BP control after 6 months.	Chronic disease self-management program; hypertension
Oh et al [34]	Significant reduction in body fat mass (*P*=.04) and HbA1c (*P*=.03) in the integrative mHealth group.	No significant changes in body weight, BMI, blood pressure, or HbA1c.	Integrative mHealth platform; hypertension and diabetes
Sittig et al [55]	Statistically significant improvements in self-efficacy (*P*=.008) and exercise (*P*=.01) in high and mid-users.	No significant differences across groups in overall health measures (ANOVA).	“capABILITY” app; diabetes
Patnaik et al [68]	Significant improvements in weight, BMI, waist circumference, and systolic blood pressure (*P*<.05).	No significant difference in metabolic equivalent (MET) levels (*P*=.54).	A mobile interactive platform: an Android-based application; diabetes
Jia et al [69]	Significant improvement in HbA1c (*P*<.001) and ABC control (*P*=.025).	No effect on hypoglycemia or weight gain (*P*=.95).	Graded the ROADMAP app and a website; diabetes
Lear et al [49]	Fewer hospitalizations and in-hospital days (*P*<.05).	No significant reduction in hospitalization rates (*P*=.12).	An internet-based self-management and symptom monitoring program for diabetes, heart failure, ischemic heart disease, chronic kidney disease, or chronic obstructive pulmonary disease
Gray et al [50]	Participants set meaningful self-management goals.	No significant improvement in quality of life (*P*=.24).	Electronic patient-reported outcome mobile app and portal system; multiple chronic diseases such as arthritis, asthma, and hypertension
Kryger et al [27]	Significant reduction in urinary tract infections (*P*=.03).	No significant change in psychosocial outcomes.	iMHere mHealth system; spinal cord injury
Gong et al [45]	Significant improvement in quality of life (*P*=.04).	No significant change in HbA1c (*P*=.83).	My Diabetes Coach program, an app-based interactive embodied conversational agent; diabetes
Chhabra et al [29]	Significant improvement in disability (*P*<.001).	No significant change in pain.	Snapcare app; low back pain
Puig et al [28]	High patient satisfaction (85.2% find it useful, 91.4% would recommend it).	No significant change in quality of life or clinical outcomes.	+Approp app; HIV
Dale et al [54]	Significant improvements in medication adherence (*P*=.004).	No long-term effects on lifestyle changes (*P*=.13).	An mHealth-delivered comprehensive cardiac rehabilitation program called Text4Heart for coronary heart disease
Dorsch et al [31]	Improvement in Minnesota Living with Heart Failure Questionnaire at 6 weeks (*P*=.04).	No effect on self-reported HF management (*P*=.78).	ManageHF4Life app; heart failure
Velardo et al [33]	High compliance with self-monitoring (96% of symptom diaries completed).	No significant impact on patient outcomes or disease progression.	“Self-management and support programme (EDGE);” COPD^f^
Poppe et al [42]	Improvement in physical activity in some groups (*P*<.05).	Limited improvements in sitting time and moderate physical activity (*P*=.09 to *P*=.07).	MyPlan 2.0 comprises a website and an optional mobile app for diabetes
Schnall et al [37]	Significant improvement in 5 symptoms (anxiety, depression, neuropathy, fever or chills, and weight loss) (*P*<.05).	No effect on other symptoms.	mobile video information provider app; HIV
Morcillo-Muñoz et al [59]	Improvement in catastrophizing, rumination, and quality of life (*P*<.05).	No effect on magnification or satisfaction with health.	NO+Dolor (NO+ Pain) app; chronic pain
Park et al [60]	Improved self-care behavior (*P*=.01) and physical activity.	No improvement in self-efficacy for managing dyspnea. The number of steps per day did not significantly differ at 6 months.	Smartphone app-based self-management program; COPD

a BP: blood pressure.

b HbA1c: hemoglobin A1c.

c ABC: the "ABC" goals for type 2 diabetes management and stands for A1C (a measure of blood sugar), blood pressure, and cholesterol (specifically low-density lipoprotein cholesterol).

d LDL-C: low-density lipoprotein cholesterol.

e mHealth: mobile health.

f COPD: chronic obstructive pulmonary disease.

mHealth technologies are emerging as a promising solution in managing chronic conditions, offering patients a convenient and efficient way to monitor and improve their health. These randomized controlled studies (n=26) that reported favorable outcomes indicate that the impact of the mHealth intervention is evident in chronic disease management [21,22,24,27-29,31,33,34,37,41,42,45,49,50,52,54,55,59,63-69]. The clinical improvements were the more consistent outcome across many of the included studies. Out of 26 RCTs, 30.8% (n=8) reported favorable improvements in key clinical health metrics, including BP [22,65,66,68], hemoglobin A1c (HbA1c) levels [21,34,41,65,69], weight [22,68], and blood glucose levels [65]. For example, the SMARTDiabetes trial [41] demonstrated better glycemic control in the intervention arm, improving HbA1c, BP, and low-density lipoprotein cholesterol control. These improvements were particularly notable in chronic diseases like diabetes and hypertension, suggesting that mHealth interventions can be effective in helping patients manage these conditions over time.

Beyond symptoms and clinical improvements, many studies found that these mHealth apps could create better self-management; 12 out of 26 studies (46%) demonstrated that patients became more involved in managing their health by tracking symptoms, medication adherence, and healthier behavior changes, such as increased physical activity [22,24,31,33,37,41,42,45,52,55,67,68]. For example, in the bipolar disorder study [64], participants using the smartphone-based self-management intervention showed reduced depressive symptoms and improved relational quality of life. This underlines the fact that mHealth tools are instrumental in improving clinical outcomes and empowering patients to take greater control of their conditions.

A common theme from these studies is increased patient engagement, which is favorable in 14 (54%) of these studies [22,24,31,33,37,41,42,45,52,54,55,63,67,68]. Most participants shared that engaging in mobile apps to track symptoms and remind themselves about their medication and educational content increased patients’ interest in their health. In the study conducted on rheumatoid arthritis, it was observed that patients who were exposed to the mobile app had better control of their disease, while the smart system of disease management group [63] had a higher rate of patients with controlled disease than the control group, at 71% versus 64.5%.

Besides engagement, user satisfaction also appeared consistently high across the positive-outcome studies. Specifically, participants expressed appreciation for the ease of use in navigating the interfaces, personalized feedback, and easy access to health care information in studies by Xu et al [24] and Puig et al [28]. For example, in the study on the Alfalfa App [24], there was improved medication adherence among patients, *P*<.001, and a very high satisfaction regarding the app’s utility in managing anticoagulation therapy.

Another important insight from the positive studies is the reduction in disease symptoms, reported in 5 of the 26 RCTs (19.2%) [27,31,37,63,64]. Indeed, many studies reported significant improvements in specific symptoms such as pain, depression, anxiety, and fatigue. This was especially true in conditions like HIV, where the mobile video information provider app [37] helped alleviate symptoms, such as neuropathy, anxiety, and depression, while also increasing medication adherence.

mHealth interventions also helped most patients improve their overall quality of life. Many studies have shown evidence for the above fact as the common resultant factor in the case of chronic diseases. Statistical improvements in scores over health-related quality of life were noticed to be significantly higher among app users than control subjects in the “My Diabetes Coach” study [45]; thus, providing evidence that such tools are effective in managing not only the clinical symptom improvement but also in emotional function and life satisfaction improvement.

In addition to clinical outcomes and user satisfaction, these mHealth interventions brought positive behavioral changes. Many studies reported that participants became more physically active, followed exercise routines more consistently, and had healthier dietary habits (11/26, 42%). For example, in a diabetes study [68], systolic BP, body fat, and BMI decreased significantly (*P*<.001) among the intervention group, thus indicating the effectiveness of mobile apps in bringing about healthier lifestyle changes.

Finally, 1 study [59] examined the cost-effectiveness of mHealth interventions. It noted that these tools are effective and economically viable, presenting affordable solutions for managing chronic diseases, especially in resource-constrained settings. For example, the chronic pain therapy study [59] established that app-based mobile treatments for pain management were effective and cost-effective, incorporating them into existing treatment plans.

### Outcomes of the Formative Studies

The following sections present the findings of the formative studies (n=42) [20,23,25,26,30,32,35,36,38-40,43,44,46-48,51,53,56-58,61,62,70-88]. The most frequent outcomes are patient engagement and satisfaction, but also clinical improvements and self-management behavior are the focus of many studies. There are some usability issues and challenges reported in the studies (Table 5).

**Table 5.** Summary of the outcomes and challenges mentioned in the formative studies (n=42).

Outcomes (favorable, no effect, and challenges)	Explanation	Frequency
Favorable outcomes		
Patient engagement and satisfaction (eg, ease of use and positive feedback) [30,32,38-40,44,46,51,58,61,70-78,88]	Most of the studies reported a high level of patient engagement and satisfaction, naming ease of use and personalized feedback as major advantages. Many studies have shown that user-friendly digital health tools improved adherence to health regimens and helped patients manage their diseases more effectively.	22
Clinical improvements (eg, pain reduction, improved blood pressure, and LDL-C^a^ levels) [25,32,39,40,43,71,79,80]	These studies reported various clinical improvements, including pain reduction, blood pressure control, improvement in LDL-C level, and overall disease management. Key results included highlighting digital health tools’ potential to enhance self-management, support patients to achieve clinical goals, and improve overall health.	8
Self-management behavior (eg, adherence to medication and lifestyle changes) [32,38-40,43,44,46,51,53,56,61,70-73,75,78,81,82]	Overall, digital health tools across these studies helped improve self-management behaviors in medication adherence, lifestyle changes, and engagement in physical activity. The most significant improvements were seen in the management of chronic diseases like COPD^b^, diabetes, hypertension, and heart failure.	19
Remote monitoring and resource usage (eg, reduction in hospital visits and health care costs) [25,32,43,51,71,83]	These studies highlight how remote monitoring systems can avert hospital admissions and reduce health costs by helping patients manage their conditions at home and, in real-time, provide the clinician with timely interventions. These findings were seen in heart failure, asthma, COPD management, hypertension, and ankylosing spondylitis, showing how digital health tools could improve clinical outcomes and optimize the use of resources within health care systems.	6
No effect		
No effect on disease management (eg, no improvement in symptom control or disease management) [30,38,48,70,79,84]	Some studies did not find any significant improvement in disease management, especially when the digital health tools did not sufficiently help engage patients or when the patients had barriers such as a lack of interest or limited use of digital health tools. In some cases, the personalization or customization of the tools was insufficient, leading to low effectiveness in managing the conditions.	6
Challenges encountered		
Usability issues (eg, technical issues and user disengagement) [32,35,38,39,61,72,88]	The most common usability issues reported across studies included technical problems, such as device inaccuracies, data syncing issues, and interface complexity. User disengagement was also another common challenge in many instances due to a lack of motivation, the tediousness of the process, and issues relating to poor integration into existing healthcare workflows.	7
Challenges in provider integration (eg, issues with workflows and data sharing) [32,38,43,44,61,71,77,78,81,83]	Provider integration challenges were highly reported in many studies, especially on integrating digital health tools with clinical workflows and the sharing of data between patients and providers. In most cases, the difficulty in adopting digital health tools in routine clinical care was cited as a barrier to clinical decision-making, with issues such as data synchronization and interoperability, assuring that providers can use the data collected remotely efficiently.	10
Privacy and data security concerns (eg, data interoperability and concerns about privacy) [32,38,43,44,61,71,76,81,85]	Privacy and data security issues were consistently identified in the reviews, particularly regarding transmission, storage, and interoperability with other healthcare information systems. There were issues of patient consent, data sharing, and following regulatory policies such as the Health Insurance Portability and Accountability Act (HIPAA). A concern was raised about protecting sensitive health information from unauthorized access.	9
Lack of participation (eg, limited use due to lack of time, motivation, or technical issues) [32,38,43,44,46,51,56,61,70-75,78,81,82]	Several of these studies repeatedly mentioned problems of non-participation for which technical issues, such as malfunction of a device and connectivity problems, together with a lack of motivation, were major reasons for dropouts and inconsistent use of digital health tools. Time constraints were also a significant factor in disengagement, as the patients struggled to integrate such tools into daily life. Personalization and support appeared very pivotal for long-term engagement.	17

a LDL-C: low-density lipoprotein cholesterol.

b COPD: chronic obstructive pulmonary disease.

One of the significant trends observed across the formative studies is the potential of digital health tools to improve the management of chronic conditions such as COPD, hypertension, diabetes, and heart failure. A significant percentage of studies (17/42, 40%) reported favorable clinical and self-management outcomes, including improved BP control, weight management, and self-efficacy. For example, HbA1c was reduced by approximately 0.79% [57], and patients suffering from COPD on the Wellinks mHealth platform showed improved symptoms and quality of life [71]. Similarly, 1 study [36] demonstrated that digital management tools for hypertension reduced systolic BP/diastolic BP by 14/5 mm Hg. These results suggest that digital tools can offer tangible improvements in managing chronic diseases, particularly when integrated with traditional care methods.

The second trend to emerge from these studies is the centrality of user-centered design in the overall success of digital health interventions. A substantial proportion of studies (13/42, 31%) indicated that designing the digital tool for patient preferences and needs enhances engagement and satisfaction. For example, the iMHere 2.0 system, which offers personalized app modules to support various self-management tasks, was praised for its customizability and ability to keep patients engaged [61]. Similarly, the Wellinks mHealth platform for COPD was well-received due to its ease of use and support in daily disease management [71]. The above findings point out the importance of developing digital tools that are not only functional but can also be tailored according to the needs of the patients in improving usability and increasing engagement.

Whereas the initial engagement and clinical outcomes from the studies were generally good, the longer-term health outcomes tend to be more mixed. The main challenges with the long-term maintenance of digital interventions were mentioned in 12% (5/42) of the selected studies, where initially engaged patients stopped using the tools due to various barriers, such as motivational issues, technical problems, or difficulties in maintaining regular use. For example, some patients in the studies of COPD management dropped off after an initial burst of engagement due to difficulties in integrating the technology into their daily routines [71]. This points to the need for continuous engagement strategies and more user-friendly designs to maintain patient involvement over the long term [26].

The studies also indicate an increased awareness that, in treating chronic conditions of a complex nature, it is more often than not challenging to rely on one-size-fits-all approaches. Some studies with generalized tools showed positive outcomes; others (5/42, 12%) indicated that tools must be customized to meet individual patient needs. For instance, a digital health tool for diabetes showed promising results in improving medication adherence but struggled with user engagement in the long term, particularly among patients who required more personalized support [39]. Many studies emphasized the need for adaptive technologies that can adjust to the changing needs of patients and those that can integrate seamlessly into existing health care systems.

Despite promising results, the need for further research and development is a constant note in several studies, as shown by 9.52% (4/42). These studies have shown that while the performance of digital tools has a promising side, there are serious gaps in personalization, scalability, and integration into health systems [60,62]. Issues of provider workflow problems [38], data interoperability [38], and assurance about patient privacy concerns were considered the most important to resolve to make them more acceptable.

## Discussion

### Principal Findings

This review highlights the growing potential of digital platforms in enabling both self-management (patient-facing monitoring, decision support, and behavior change) and provider-led management (remote monitoring dashboards, care coordination, and clinical decision support). The digital platforms were primarily designed for use at home and complement patients’ routine health care practices seamlessly, giving major importance to self-monitoring, personalization, and motivational aspects such as rewards. While self-management is valued, our findings suggest that platforms without embedded communication facilities with health care professionals or social support may limit user interaction and effectiveness. The fact that collaborative care and self-management co-occurred in the included studies underscores the value of hybrid interventions that combine patient autonomy with professional oversight.

A significant proportion of the studies reported the usage of co-design or user-centered design approaches as the best practice when developing health interventions. The lack of methodological details and theoretical underpinnings in some studies hinders clear conclusions regarding their effectiveness. While the review found a range of features, there was limited evidence on which features are most effective at facilitating long-term engagement. Most studies were feasibility or pilot studies with brief follow-up periods and small or nonrandomized samples. As such, large-scale assessments of the effectiveness of platforms, particularly for long-term health outcomes, are lacking. Platforms addressing single chronic diseases were more likely to exhibit improved outcomes, whereas those addressing multimorbidity encountered more usability and implementation issues, underscoring the need for more personalized designs to meet complex health needs.

One of the significant barriers to the long-term adoption of digital health solutions was user disengagement. Several longitudinal studies reported high dropout rates due to a lack of motivation or technical issues, highlighting the importance of adaptive and personalized engagement strategies that are unobtrusive and seamlessly integrate digital health platforms into everyday life.

In the following sections, we elaborate on the principal findings and compare and contrast them with relevant literature.

### Characteristics of Digital Platforms Supporting the Management of Chronic Diseases

Our findings show that most of the interventions focused on self-management of chronic diseases. We also found that the vast majority of interventions were designed to be used at home by participants, usually in conjunction with their ongoing health care plans. Self-management is critical in managing chronic disease [94,95], and new digital platforms heavily target this aim. According to the literature, chronic disease can be managed well by balancing traditional medical care with self-management [96]. Effective self-management requires optimal communication with health care teams [97]. Although self-management requires support from health care providers and caregivers, this review discovers that not all identified platforms offer such options. Similarly, it is necessary that digital platforms provide options for social support to maintain long-term engagement in self-managing disease [98]. Only a few platforms in our review had options for social support. Our findings suggest that features like self-tracking, customization, and rewards support users’ engagement with digital platforms [12]. Although we have identified a range of features in the studied platforms, there is still a lack of evidence in the included studies as to which features are best for supporting long-term engagement. Therefore, more research is needed to investigate which features of digital platforms will best support long-term user engagement and motivation.

The findings from applying the NICE Evidence Standards Framework emphasize the necessity of a multifaceted and integrated approach to delivering effective digital health interventions. The significance of self-management reflects the growing emphasis on patient empowerment, enabling individuals to actively monitor, track, and manage their health. However, self-management alone is not always sufficient, as structured support systems improve engagement and adherence. The frequent co-occurrence of self-management with collaborative care (47 times) highlights the effectiveness of a hybrid model that combines patient autonomy with a form of clinical oversight. This integration ensures that while patients take a leading role in their health management, they are still supported by health care professionals who provide guidance and add to the rigor of the digital intervention. However, as noted earlier, some platforms lack direct communication with health care providers, which could reflect as a limiting factor in their effectiveness as a digital intervention. This comprehensive analysis highlights the interdependencies among digital intervention strategies, advocating for a cohesive, patient-centered approach. Self-management is most effective when complemented by collaborative care, education, and evidence-based tools, ensuring engagement, clinical effectiveness, and long-term sustainability in chronic disease management.

### Principles and Frameworks Used in Designing and Developing the Platforms

We have observed that more than half of the studies reported some form of co-design, consultative, or user-centered approach to the development of digital platforms. This is a positive indication that health care interventions are co-developed to meet the needs of the stakeholders, as suggested in the literature [99,100]. Overall, adopting a co-design approach might have contributed to positive outcomes. However, due to the lack of relevant data in those studies, we could not determine if there was any relationship between the co-design of the digital platforms and their effectiveness in managing chronic disease. A lack of theoretical basis in some of the included studies limits their reliability. Therefore, it is suggested that the future development of digital platforms needs solid theoretical support, and such support may well improve effectiveness and user engagement.

A range of behavior-change techniques was used in the included studies, generally informed by behavior-change models and social cognitive theory [101]. However, future studies should focus on measuring the effects of these design principles, determining the extent to which they contribute to the efficacy and continued use of the platforms. In addition, we identified 5 platforms that used gamification techniques to enhance user experience and embed learning principles. This suggests that platform designers considered the notion that gamification improves health behaviors, as reported in the literature [102].

### Effectiveness and Efficacy of the Platforms

Most of the included studies were pilot in nature, focusing either on design, development, usability, uptake, or clinical utility. In many of these studies, not all features of the platforms were tested for effectiveness. One key reason for not testing all the features of a digital platform during the trial period (for example, study by Doyle et al [44]) was that the development was based on the initial success of the trial, and this was the focus of the majority of the included studies. This suggests that future work should identify those studies where the full potential of digital platforms is evaluated, so that more complete conclusions can be drawn about the platforms’ effectiveness.

It is important to note that the majority of studies reporting improved health outcomes focused on a single or localized chronic disease. Conversely, those reporting no effects on health outcomes largely focused on multiple chronic diseases. This suggests that focused digital platforms have a higher likelihood of positive health outcomes than those targeting multiple chronic health issues. Further research is needed to investigate how digital platforms could successfully be designed and evaluated to manage multimorbidity (ie, the presence of 2 or more long-term health conditions). Furthermore, it is worth noting that the testing duration of the digital platforms was generally short in most studies. In some cases, the formal sample size was not calculated since the studies were feasibility or pilot studies. This highlights the importance of meticulously evaluating digital platforms with appropriate sample sizes in future studies to ensure the validity and reliability of the research.

### Uptake of the Digital Health Interventions

A few of the included studies, which were conducted over a longer period (ie, 6 months or more), explicitly mentioned dropout rates and the causes of disengagement, including lack of motivation, technical issues, or health problems. For example, a study [86] aimed to understand the effects of long-term (eg, 12 months) smartphone-based self-monitoring in patients with lipid metabolism disorders reported that 43% (43/100) of patients never started using the app due to a lack of time, health problems, lack of motivation, and technical problems. Dropout due to technical issues (eg, poor wireless connection) was also reported in another study, where an mHealth system was developed for managing chronic conditions [61], where 1 patient dropped out of the study after the initial engagement. Another included study [39] mentioned that approximately 80% (400/500) of users used Mobiab (ForaCare Suisse AG) for managing diabetes for less than 1 week. Such a dropout may imply that the daily use requirements of the app were challenging for individuals to maintain. User fatigue is a likely issue where daily data entry or constant interaction with digital tools is required. As digital health interventions often require continuous self-monitoring and engagement—whether through tracking symptoms, inputting data, or responding to feedback—users may experience burnout, leading to disengagement. A high dropout rate (95/162, 58.6%) was mentioned in a study [66] where the effect of an mHealth self-monitoring intervention among black individuals with uncontrolled hypertension was tested for 1 year. While digital health tools show promise in improving short-term health outcomes, dropout rates, user fatigue, and sustainability remain substantial challenges. For digital health interventions to be successful in the long run, continuous engagement strategies and adaptive features must be prioritized to ensure users remain motivated and that interventions can be integrated smoothly into everyday life. However, further investigation is required to understand the dropout and disengagement with digital health interventions.

Digital literacies play a key role in the uptake of digital health interventions [103]. Researchers found that even though patients with low literacy may have access to technology, they may not be able to use it without any help [104]. Some studies were conducted with adults aged between early (20‐39 y) [27,35,43,61] and middle adulthood (40‐59 y) [30,34,40,55]. Chronic diseases are more common among older adults, who may have poor digital literacy and difficulty adopting new information technologies. In the included studies, no information was collected regarding the digital literacy levels of the participants. However, some of the included studies had information about income and education level that are linked to the participants’ digital skills and health literacy. Low digital literacy may be a barrier to the adoption and engagement of digital platforms. Another limitation of the included studies is that not all studies reported parameters such as participants’ skills, experience, or level of education, and these might have contributed to the infrequent use of the platform [28] or withdrawal from the study [42]. Some of the studies excluded patients who were unwilling to participate or could not meet the study requirements. Such nonparticipants may provide insights for the design of more user-centered platforms. Therefore, future studies should account for the varying digital literacies of different cohorts of platform users, as this may impact the overall feasibility of digital health interventions.

Participant confidentiality, including data security, was not widely discussed in the papers included in this review. Only 1 paper provided a detailed overview of the data security measures of their platform [39]. Researchers have reported that health and fitness apps often violate users’ privacy by not following existing guidelines and regulations [105,106]. Therefore, it is of the utmost importance to protect the health data that digital platforms gather. Ensuring the privacy and security of health data may contribute to the long-term uptake of digital health interventions.

### Implications and Real-World Adoption

While most studies focus on the efficacy of digital health interventions in pilot settings, large-scale integration within already prevailing health care systems was not reported in any studies. However, some studies have promising steps toward integration within the current health care system. As an example, a mobile app called Mobiab [39] for diabetes management integrated with health care systems was partially successful, particularly where patient data could be automatically transmitted to clinicians through platforms like mobile apps. The study reported that the use of different software was an additional complication for the clinicians, as they already used some commercial software. Also, the researchers did not implement data into hospital information systems due to the lack of communication interface specifications. A hypertension management platform was developed that can be integrated into EHRs to facilitate real-time patient data analysis and effective decision-making [85]. However, the efficacy of the system is yet to be tested. Another application called “electronic patient-reported outcome mobile app and portal system” was developed for people with complex care needs. During the trial, the electronic patient-reported outcome system was not interfaced with other existing technology systems, that is, EMRs or other available platforms, but the system was designed in such a way that interoperability could be a possibility [50].

None of the studies explicitly discusses interoperability, clinician workload, or regulatory constraints, although these could hinder the implementation of digital health solutions in real-world health care settings [107,108]. These barriers are implicit in the included studies and require investigation in future studies. For example, one of our included studies [39] refers to the long-term involvement of clinicians in managing diabetes but does not discuss the potential impact it would have on clinician workload. Clinicians would be required to review the data generated by the app, resulting in an increased workload if the app is not providing actionable insights or is not well-integrated into clinical workflows. Research shows that the lack of seamless integration of digital health platforms into health care systems poses obstacles to broader adoption and implementation [109,110] and may hinder sustainability and scalability. In addition, regulatory restrictions on patient data protection (eg, General Data Protection Regulation and Health Insurance Portability and Accountability Act) are not discussed in the included studies, but these would be necessary to make the platform health care regulation compliant and legally viable for real-world adoption and implementation. Such concerns were echoed in a recent study, where authors highlighted the need for transparent data government policies to be implemented in order to meet regulatory requirements and address security concerns [107].

Many of the papers included in this review suggested that further research into the interventions was needed. Reasons for this included limited sample sizes, limited participant uptake, technical issues, and the need for further personalization of the platforms. Furthermore, while short-term health outcomes of the digital interventions were largely positive, long-term outcomes remain generally unknown. Thus, it was frequently suggested that strategies to maintain long-term use were required, along with further analysis of platform use. Additionally, it was noted in some instances that future studies should account for the varying digital literacies of different cohorts of users, as this may impact the overall feasibility of digital health interventions. Finally, where studies focused on a single or localized chronic health issue, it was broadly concluded that platform design could easily be replicated to address other health issues. However, interventions that sought to address multiple chronic diseases had a higher incidence of technical issues or problems with usability and feasibility. This could be because those studies have methodological challenges, such as higher sample sizes and complex sampling frames to measure the required outcomes. However, such challenges were not reported in those studies, and this suggests that further research is needed to enable digital health interventions to effectively address multiple chronic health conditions.

### Limitations

There are several limitations to our study. Our objective was to explore the broader perspective of chronic diseases rather than focusing on individual chronic diseases. Therefore, the search strategy used was general terms related to chronic disease management rather than specific conditions such as diabetes, hypertension, cardiovascular disease, or COPD. While this approach allowed us to draw on a wide range of interventions, it may have inadvertently omitted condition-specific intervention studies using narrowly defined keywords. This could have affected the completeness of the review in 2 ways. First, we might have missed highly specialized interventions tailored to the unique management needs of particular chronic diseases. Second, excluding disease-specific search terms could have led to underrepresenting certain populations or technologies in specific disease domains. Nonetheless, studies in this review constitute an important and representative sample of the current digital intervention landscape for chronic disease management. These studies encompass a diverse range of technologies, user groups, and interventions to identify trends, design issues, and gaps in digital interventions for chronic disease management. We acknowledge that a more targeted search strategy, perhaps in a future scoping or systematic review, would offer more information about condition-specific digital health innovations and their impact.

Our search strategy did not include the exact keyword “self-management” or corresponding controlled-vocabulary terms. Although the broader “management” concept and citation chasing were used to capture patient-led self-management studies indirectly, records that exclusively use “self-management” terminology may have been missed. A future update should incorporate a self-management term cluster (eg, “self-management,” “self care,” and “self-monitor”) and mapped controlled vocabulary to improve sensitivity.

In this review, we only included articles that were published in English. For the included studies, we have primarily reported the results qualitatively. Where available, we reported the frequency of outcomes but were unable to capture the effect size due to the variability of the studies. Furthermore, not all studies measured the impact of digital platforms; several of them instead measured the usability and acceptability of those platforms. Therefore, we could not compare all the outcomes, and in some cases, the outcomes were inconclusive due to the preliminary nature of the studies. Finally, the digital platforms included in the studies were designed for diverse users with varying degrees of digital literacy. However, we could not analyze how the digital literacies of the participants contributed to outcomes because such data were not reported in the studies.

### Conclusions

This study provides a comprehensive overview of digital platforms for managing chronic diseases, delineating features for self-management versus provider-led management. Overall, the vast majority of papers in this review concluded that digital health interventions can be beneficial in managing chronic health issues. They also indicated that the adoption of such methods in combination with regular clinical care has the potential to improve health outcomes, support self-management, and support communication between patients and health care providers. However, challenges remain in long-term engagement, overcoming technological barriers, and integrating these tools into existing workflows in health care. The effectiveness and acceptance of digital health interventions vary based on patient characteristics, such as age, health literacy, and the capacity for intervention tailoring. Success will be contingent on interventions that can fulfill specific patient needs through user-centered, tailored engagement while being effortless to use and integrated seamlessly within the health care ecosystem. These tools, therefore, require further research for their full development so that they are adaptable, scalable, and meet the diverse needs of patients with chronic conditions. More research is needed to further develop these tools for wider acceptance and improving engagement.

## Supplementary material

10.2196/63742 Multimedia Appendix 1 Database search outcome.

10.2196/63742 Multimedia Appendix 2 Chronic diseases reported in the included studies (n=83).

10.2196/63742 Multimedia Appendix 3 Digital intervention strategies, features of the digital platforms, and co-occurrence matrix of the digital intervention strategies.

10.2196/63742 Checklist 1 PRISMA-ScR checklist.

## Abbreviations

AI: artificial intelligence
BP: blood pressure
COPD: chronic obstructive pulmonary disease
EHR: electronic health record
EMR: electronic medical record
HbA1c: hemoglobin A1c
mHealth: mobile health
NICE: National Institute for Health and Care Excellence
PRISMA-ScR: Preferred Reporting Items for Systematic reviews and Meta-Analyses extension for Scoping Reviews
RCT: randomized controlled trial
