# Supplementary material for: Digital Health Interventions to Support Chronic Disease Management: Systematic Scoping Review
Source: JMIR Mhealth Uhealth. 2026 Jan 14;14:e63742. doi: 10.2196/63742 (PMC12803440; doi:10.2196/63742)
Supplement: Multimedia Appendix 1 [file mhealth-v14-e63742-s001.docx]

**Appendix 2: Search outcome (2013-2024)**

| **Database** | **Search string** | **Number of records identified** |
| --- | --- | --- |
| **PsycInfo**  **(2013-2022)**  **(n=382)** | "chronic disease" or "chronic illness" or "long term conditions" or "chronic conditions"  AND  "Digital" or "mHelath" or "App"  AND  "management" AB ("chronic disease" OR "chronic illness" OR "long term conditions" OR "chronic conditions" ) AND AB ( "Digital" OR"mHealth" OR "APP" ) AND AB "Management" | 244 |
|  | "chronic disease" AND "Digital Tech" AND "management" | 0 |
|  | "chronic disease" AND "Digital" AND "management" | 59 |
|  | "chronic disease" AND "mHealth" AND "management" | 43 |
|  | "chronic disease" AND "app" AND "management" | 36 |
|  | **Total** | **382** |
| **2022-2024**  **(n=47)** | "chronic disease" AND "Digital Tech" AND "management" | 0 |
|  | "chronic disease" AND "Digital" AND "management" | 33 |
|  | "chronic disease" AND "mHealth" AND "management" | 8 |
|  | "chronic disease" AND "app" AND "management" | 6 |
|  | **Total** | **47** |
| **Scopus**  **(2013-2022)**  **(n=1001)** | “chronic disease” AND “Digital Tech” AND “management” | 0 |
|  | “chronic disease” AND “Digital” AND “management” | 381 |
|  | “chronic disease” AND “mHealth” AND “management” | 274 |
|  | “chronic disease” AND “App” AND “management” | 346 |
|  | **Total** | **1001** |
| **2022-2024**  **(n=591)** |  |  |
|  | "chronic disease" AND "Digital Tech" AND "management" | 0 |
|  | "chronic disease" AND "Digital" AND "management" | 308 |
|  | "chronic disease" AND "mHealth" AND "management" | 115 |
|  | "chronic disease" AND "App" AND "management" | 168 |
|  | **Total** | **591** |
| **Web of Science**  **(2013-2022)**  **669** | "chronic disease" AND "Digital Tech" AND "management" | 0 |
|  | "chronic disease" AND "Digital" AND "management" | 268 |
|  | "chronic disease" AND "mHealth" AND "management" | 219 |
|  | "chronic disease" AND "App" AND "management" | 182 |
|  | **Total** | **669** |
| **(2022-2024)**  **(n=250)** |  |  |
|  | "chronic disease" AND "Digital Tech" AND "management" | 0 |
|  | "chronic disease" AND "Digital" AND "management" | 153 |
|  | "chronic disease" AND "mHealth" AND "management" | 45 |
|  | "chronic disease" AND "App" AND "management" | 52 |
|  | **Total** | **250** |
| **IEEE Xplore (2013-2022)**  **(n=5)** | "chronic disease" AND "Digital Tech" AND "management" | 0 |
|  | "chronic disease" AND "Digital" AND "management" | 3 |
|  | "chronic disease" AND "mHealth" AND "management" | 1 |
|  | "chronic disease" AND "App" AND "management" | 1 |
|  | **Total** | **5** |
| **(2022-2024)**  **(n=3)** | "chronic disease" AND "Digital tech" AND "management" | 0 |
|  | "chronic disease" AND "Digital" AND "management" | 3 |
|  | "chronic disease" AND "mHealth" AND "management" | 0 |
|  | "chronic disease" AND "App" AND "management" | 0 |
|  | **Total** | **3** |
| **Medline (via Ovid)**  **(2013-2024)**  **(n=442)** | "chronic disease" AND "Digital tech" AND "management" | 0 |
|  | "chronic disease" AND "Digital" AND "management" | 232 |
|  | "chronic disease" AND "mHealth" AND "management" | 111 |
|  | "chronic disease" AND "App" AND "management" | 99 |
|  | **Total** | **442** |
|  |  |  |
| **Embase**  **(2013-2024)**  **(n=10002)** | "chronic disease" AND "Digital tech" AND "management" | 0 |
|  | "chronic disease" AND "Digital" AND "management" | 576 |
|  | "chronic disease" AND "mHealth" AND "management" | 179 |
|  | "chronic disease" AND "App" AND "management" | 247 |
|  | **Total** | **10002** |
