# Supplementary figures and images for: Digital Health Interventions to Support Chronic Disease Management: Systematic Scoping Review
Source: JMIR Mhealth Uhealth. 2026 Jan 14;14:e63742. doi: 10.2196/63742 (PMC12803440; doi:10.2196/63742)

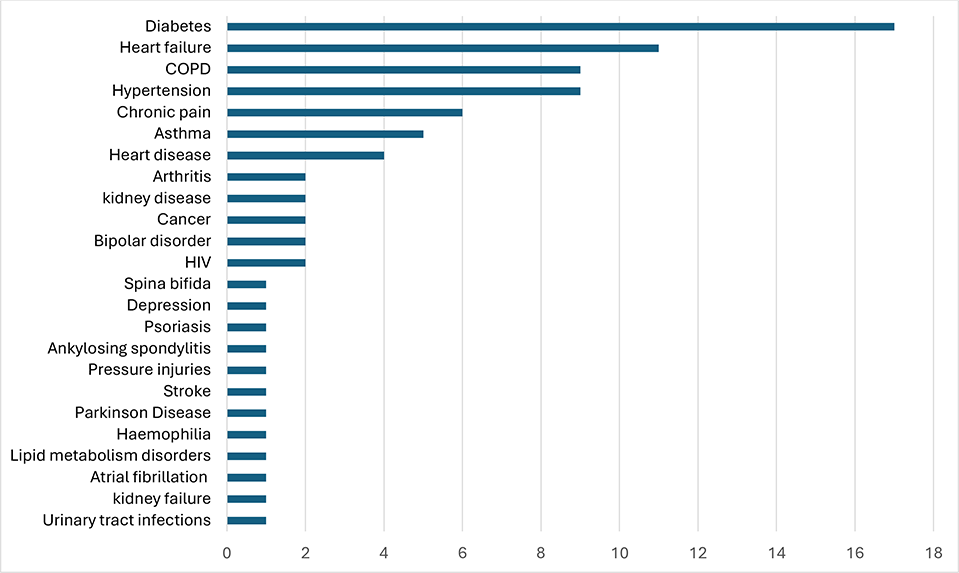

Supplement: Multimedia Appendix 2 [file mhealth-v14-e63742-s002.png]
