# Supplementary material for: Digital Health Interventions to Support Chronic Disease Management: Systematic Scoping Review
Source: JMIR Mhealth Uhealth. 2026 Jan 14;14:e63742. doi: 10.2196/63742 (PMC12803440; doi:10.2196/63742)
Supplement: Multimedia Appendix 3 [file mhealth-v14-e63742-s003.docx]

Multimedia Appendix 3: Digital intervention strategies, features of the digital platforms and co-occurrence matrix of the digital intervention strategies

Digital Intervention Strategies extracted from NICE Framework

| **Digital Intervention Strategy** | **Description** |
| --- | --- |
| Self-Management | Allow people to self-manage a specified condition. May include behaviour change techniques such a Nudges, Gamification, Cognitive Behavioural Therapy etc. This allows users to record, and optionally to send, data to a healthcare professional to improve management of their condition. |
| Digital Therapeutics (Treat) | Provide treatment or guide treatment. Treating mental health or other conditions. Clinician-facing apps that may advise on treatments in certain situations. |
| Information and Education (Inform) | Provides information, resources or activities to the public, patients or clinicians. Includes information about a condition general health and lifestyle. Describes a condition and its treatment. Apps providing advice for healthy lifestyles (such as recipes). Apps that signpost to other services. |
| Personal Health Record System (Simple Monitoring) | Includes general health monitoring using wearables and symptom diaries. Health tracking information such as from fitness wearables. Symptom or mood diaries. |
| Collaborative Care (Communicate) | Allows 2-way communication between patients and/or healthcare professionals. Instant messaging apps for health and social care. Video conference-style consultation software. Platforms for communication with carers or professionals. |
| Active Monitoring | Automatically records information and transmits the data to a professional, carers or third-party organization, without any input from the user, to inform clinical management decisions. Devices such as implants, sensors worn on the body or in the home. Data are automatically transmitted through the platform for remote monitoring. |
| Clinical Decision Support System (Calculate) | Tools that perform clinical calculations that are likely to affect clinical care decisions. Allows for clinicians and/or users to calculate parameters pertaining to care, such as early warning system software. |

Features of the digital platforms and corresponding digital intervention summary extracted from the included studies

| **Author, year** | **Target Disease** | **Platform type** | **Key Features** | **Intervention Summary** | **Digital Intervention Strategy based on**  **NICE Framework** |
| --- | --- | --- | --- | --- | --- |
| Brandl et al., 2022 | Psoriasis | Smartphone App | Medication management; Food diary; Activity tracking; Rate doctor; Patient-provider communication | A Co-designed Self-Management Tool (SMT) for patients with Psoriasis. Content Management web portal for the editorial team to maintain SMT. | Self-Management; Personal Health Record System; Collaborative Care. |
| Woo Oh et al., 2022 | Obesity, hypertension, diabetes | Smartphone App & Bluetooth-enabled self-measuring device | Activity tracking; BP*, Glucose*, Body composition*; Patient-provider communication; Patient reports. | A Bluetooth BP Monitor and Blood Glucose Monitor + LIBIT (mobile app for diet, fitness, and self-health monitoring), Mediram App (for medication). Web interface for clinician. | Self-Management; Personal Health Record System; Collaborative Care. |
| Steele Gray et al., 2016 | Complex chronic disease & disability | Smartphone App & *Web-based portal | Patient reports, Goal management; Hospital checkout. | A Patient-Reported Outcome (ePRO) system (mobile app and web portal) was designed and developed to facilitate goal-oriented care. | Self-Management; Personal Health Record System. |
| Xiang Gu et al., 2019 | Chronic heart failure | Web-based portal, Tracking device & Smartphone App | Symptom tracking; BP*; Body composition*; Patient-provider communication; Patient reports. | A hospital-community-family (HCF)–based telehealth program for patients with chronic heart failure (CHF). This program integrates smart health tracking devices, a mobile app and a portal to facilitate remote management by both general practitioners and cardiologists. | Self-Management; Personal Health Record System; Collaborative Care. |
| Sittig et al., 2020 | Diabetes | Smartphone App | Food diary; Activity tracking; Glucose*; Messaging. | A theory-driven mHealth mobile app named capABILITY, which incorporates trigger messages based on the Fogg Behavior Model (FBM) to enhance self-efficacy, knowledge, and self-care in patients with type 2 diabetes. | Self-Management. |
| Patnaik et al., 2022 | Diabetes | Smartphone App & **Website | BP*; Body composition*; Reminders. | A mobile application designed to promote physical activity among newly diagnosed type II diabetes patients. The intervention involved using the app to encourage meeting WHO recommendations for physical activity. | Self-Management. |
| Weiping Jia et al., 2021 | Diabetes | Smartphone App & Website | Patient reports, Appointment management; Patient-provider communication | A mobile health (mHealth)-enabled hierarchical diabetes management system (mobile app for patient, website for clinician) in China aimed at improving type 2 diabetes control in primary care settings. The intervention involved a tiered care team approach and monthly blood glucose monitoring, supplemented by capacity building and performance reviews. | Self-Management; Collaborative Care. |
| Lear et al., 2021 | Diabetes, heart, kidney & pulmonary diseases | Website | Symptom tracking; BP*; Glucose*; Body composition*; Patient-provider communication; reminders | A self-management program delivered through a website (internet CDM) for patients with multiple chronic diseases in British Columbia, Canada. The program included telephone nursing support and was integrated within primary care settings, compared to usual care. | Self-Management; Personal Health Record System. |
| Steele Gray et al., 2021 | Arthritis, asthma, cancer, pulmonary disease, hypertension, heart & kidney failure | Smartphone App & Web-based portal | Goal management | A Patient-Reported Outcome (ePRO) system (mobile app and web portal) was designed and developed to facilitate goal-oriented care. | Self-Management. |
| Kryger et al., 2019 | UTI, pressure injuries | Smartphone App & Web-based portal | Symptom tracking; Patient-provider communication; Patient reports; Reminders | An Interactive Mobile Health and Rehabilitation (iMHere) system, mHealth tool (mobile app and web portal) designed to support self-management for individuals with spinal cord injuries (SCI). | Self-Management; Collaborative Care. |
| Gong et al., 2020 | Diabetes | Smartphone App & Web-based portal | Glucose*; Patient-provider communication; Automated conversation agent; counselling | An app-based interactive conversational agent named Laura (My Diabetes Coach - MDC), designed to support self-management of type 2 diabetes at home over 12 months. This randomized controlled trial in Australia compared the effects of the MDC program against usual care on glycated hemoglobin (HbA1c) and health-related quality of life (HRQoL). | Self-Management; Digital Therapeutics; Information and Education. |
| Chhabra et al., 2018 | Chronic low back pain | Smartphone App | Symptom tracking; Activity tracking; Patient reports; Goal management | Assessment of a smartphone app, "Snapcare", on pain and functional outcomes in patients with chronic low back pain. | Self-Management. |
| Bailey et al., 2020 | Knee & back pain | Smartphone App & Wearable motion sensor | Activity tracking; Patient-provider communication; Instructional videos. | A digital care program (DCP) delivered via a mobile app for patients with chronic knee and back pain. This is a multimodal program that included educational content, sensor-guided exercise therapy (ET), and behavioral health support, complemented by one-on-one remote health coaching. | Self-Management; Collaborative Care; Information and Education. |
| Setiawan et al., 2019 | Chronic diseases & disabilities | Smartphone App and Web-based portal | Patient-provider communication; Patient reports; Personal treatment plan | An improvement on the initial iMHere mHealth system which includes a mobile app for the patient and caregiver, with a web portal for clinician. iMHere 2.0, is designed to support individuals with chronic conditions and disabilities (PwCCDs). This upgraded version aimed to be more adaptive and scalable, incorporating various self-management support modules tailored to the changing needs of PwCCDs. | Self-Management; Collaborative Care; Digital Therapeutics. |
| Puig et al., 2021 | HIV | Smartphone App | Patient-provider communication; Patient reports; Reminders; Counselling | A mobile app (for patient) and web interface (for health center) for HIV-infected patients aged 60 years or older. | Self-Management; Collaborative Care; Digital Therapeutics. |
| Park et al., 2020 | COPD | Smartphone App | Symptom tracking; Patient-provider communication; Personal treatment plan; Counselling | A smartphone app-based self-management program for individuals with chronic obstructive pulmonary disease (COPD). This program is guided by social cognitive theory and self-efficacy theory. | Self-Management; Collaborative Care; Digital Therapeutics. |
| Burda et al., 2022 | Diabetes | Smartphone App & Web-based portal | Food diary; Activity tracking; Glucose*; Patient-provider communication | Mobiab is a mobile and web app designed for long-term diabetes mellitus (DM) management in the Czech Republic. This system improves DM tracking by replacing traditional paper-based methods with customizable modules that adapt to user preferences. | Self-Management; Collaborative Care. |
| Parmanto et al., 2013 | Spina bifida | Smartphone App & Web-based portal | Symptom tracking; Patient reports; Reminders; Appointment management | The project focused on developing and implementing a novel mobile health (mHealth) system called iMHere (iMobile Health and Rehabilitation) to enhance care for individuals with spina bifida and other chronic conditions. This system integrated smartphone apps and a clinician portal connected through a secure two-way communication protocol. | Self-Management; Collaborative Care. |
| Evans et al., 2016 | Heart failure | Telehealth & Wireless watch | Activity tracking; BP*, Body composition* | A telehealth system for older adults, both with and without heart failure. Participants used a wireless wristwatch-based monitoring device to continuously collect temperature and motion data, along with other health measures like weight, blood pressure, and daily health surveys via a weight scale, blood pressure cuff, and tablet. | Self-Management; Personal Health Record System; Active Monitoring. |
| Breckner et al., 2022 | Diabetes, COPD, high BP, heart failure | Website | Symptom tracking; BP*; Glucose*; Body composition*; Mental health (self-report); Goal management; Appointment management; Warning system | The study evaluated the TelePraCMan web app, an intervention designed for self-management of chronic diseases such as type 2 diabetes, chronic obstructive pulmonary disease, high blood pressure, and heart failure within a German primary care setting. | Personal Health Record System; Clinical Decision Support System. |
| Dale et al., 2015 | Coronary heart disease | Website | Messaging | A mobile health-delivered comprehensive cardiac rehabilitation (CR) program, Text4Heart, aimed at improving adherence to multiple healthy lifestyle behaviors (smoking cessation, physical activity, healthy diet, and nonharmful alcohol use) among New Zealand adults diagnosed with coronary heart disease (CHD). This intervention included personalized, social cognitive theory-based daily SMS messages and a supporting website, in addition to traditional center-based CR. | Self-Management. |
| Salari et al., 2021 | Diabetes | SMS & Website | Food diary; Activity tracking; Glucose*; Patient reports; Messaging | This study focused on the development and usability evaluation of a Transtheoretical model (TTM) based, cloud and mobile-based diabetes self-management app, aimed at facilitating health behavior changes and enabling remote monitoring by healthcare providers for people with type 2 diabetes. | Self-Management; Collaborative Care. |
| Xiaojian et al., 2019 | Ankylosing Spondylitis | Smartphone App & Web-based portal | Symptom tracking; Medication management; Patient reports; Appointment management; | A "Smartphone SpondyloArthritis Management System" (SpAMS), an interactive mHealth tool, to manage ankylosing spondylitis (AS) and other forms of spondyloarthritis (SpA). This system combines patient and physician portals within a smartphone application, facilitating online self-assessments by patients and allowing rheumatologists to record assessments and treatments during clinic visits. | Self-Management. |
| Duan et al., 2020 | Hypertension | Smartphone App | Symptom tracking; Medication management; BP*; Body composition*; | A mHealth mobile app aimed at improving patient compliance with hypertension self-management, utilizing a goal-directed design method. | Self-Management. |
| Milani et al., 2017 | Hypertension | Smartphone App, Website, Wearable tech (BP monitor) | Medication management; Activity tracking; BP* | A home-based digital-medicine program for managing uncontrolled hypertension patients, comparing the outcomes with usual-care patients matched by age, sex, BMI, and blood pressure levels. The digital-medicine group participated in a 90-day program where they submitted weekly blood pressure readings online, received medication management and lifestyle recommendations from a clinical pharmacist and health coach, and used blood pressure units that automatically updated their electronic medical records. | Self-Management; Collaborative Care. |
| Dorsch et al., 2021 | Heart failure | Mobile App and Fitbit | Symptom tracking; Activity tracking; BP*; | ManageHF4Life is designed to enhance self-management in patients with heart failure (HF). The mobile app promotes daily self-monitoring and self-management. The primary focus was on changes in the Minnesota Living with Heart Failure Questionnaire (MLHFQ) scores and secondary outcomes included the Self-Care Heart Failure Index (SCHFI) scores and HF readmission rates. | Self-Management. |
| Velardo et al., 2012 | COPD | Smartphone App & Website, Wearable tech | Symptom tracking; BP*; Patient-provider communication; Appointment management; Instructional videos | A digital health system designed to aid self-management for patients with chronic obstructive pulmonary disease (COPD). This system featured a mobile application on a tablet and a secure website for healthcare professionals. | Self-Management; Collaborative Care; Information and Education. |
| Cormican & Dowling 2020 | Multiple myeloma | Website | Symptom tracking; Activity tracking | The study involved the development of a digital tool aimed at assisting patients with relapsed or refractory multiple myeloma (MM) in self-managing their condition by monitoring and managing side effects and symptoms. | Self-Management. |
| Doyle et al., 2021 | COPD, CHF, CHD, diabetes | Measurement devices, Smartphone App | Symptom tracking; BP*; Glucose*; Patient-provider communication; Personal treatment plan | A digital health platform designed to assist older adults with multimorbidity in self-managing their conditions at home (ProACT). The platform aimed to support users with multiple chronic diseases by providing integrated care tools and services, including a clinical triage service for symptom alerts and a technical helpdesk. | Self-Management; Collaborative Care; Digital Therapeutics; Clinical Decision Support System. |
| Poppe et al., 2019 | Diabetes | Smartphone App & Website | Patient-provider communication; Goal management; Personal treatment plan | An automated eHealth and mHealth intervention (MyPlan 2.0) designed to influence physical activity (PA) and sedentary behavior (SB) in two distinct populations: adults with type 2 diabetes mellitus (T2DM) and adults aged 50 years and older. The intervention was informed by the Health Action Process Approach (HAPA) and aimed to modify participants' PA and SB through a series of five weekly guided sessions. | Self-Management; Collaborative Care. |
| Gelbman & Reed., 2022 | COPD | Smartphone App | Symptom tracking; Medication management | A mHealth platform (Wellinks) for patients with chronic obstructive pulmonary disease (COPD) over an 8-week period. The platform integrated several devices such as the Flyp nebulizer, Smart One spirometer, and Nonin pulse oximeter, alongside a mobile app that facilitated daily symptom and medication tracking. | Self-Management. |
| Schnall et al., 2018 | HIV | Smartphone App | Symptom tracking; Personal treatment plan; Messaging; Instructional videos | A mHealth app, named mobile Video Information Provider (mVIP), which offers evidence-based self-care strategies for managing common symptoms experienced by people living with HIV (PLWH). | Self-Management; Information and Education; Collaborative Care. |
| Alharbey & Chaterjee., 2019 | COPD | Smartphone App | Symptom tracking; BP*; Patient reports; Instructional videos; Modules; Warning system | A mHealth app (MyLung) designed to enhance self-awareness and improve self-care management among patients with Chronic Obstructive Pulmonary Disease (COPD). The app includes three integrative modules focused on education, risk reduction, and monitoring. | Self-Management; Information and Education; Clinical Decision Support System. |
| Noviani et al., 2020 | Diabetes | Smartphone App | Symptom tracking; Patient-provider communication; Instructional videos; Messaging | The study evaluated the impact of the 'Sahabat Diabetes' mobile app, on improving self-efficacy and controlling HbA1c levels in patients with type 2 diabetes mellitus (T2DM) with uncontrolled HbA1c levels (>7). A focus placed on a Diabetes Self-Management Education (DSME) through the mobile app. | Self-Management; Information and Education; Collaborative Care. |
| Morcillo -Muñoz et al., 2022 | Chronic musculoskeletal pain | Smartphone App | Symptom tracking; Medication management; Activity tracking; Personal treatment plan; Warning system | A multimodal intervention program delivered via a mobile app for managing chronic pain in an outpatient setting. The intervention group engaged in a standard web-based psychosocial therapy program via a smartphone for six weeks, focusing on various activities designed to manage pain. In contrast, the control group had access to basic audiovisual material about pain management through the app. | Self-Management; Digital Therapeutics; Information and Education; Clinical Decision Support System. |
| Steinert et al., 2020 | Lipid metabolism disorders | Smartphone App | Symptom tracking; Medication management; Activity tracking; Body composition*; Patient reports | The study evaluated the effectiveness of a smartphone application (MyTherapy) designed to assist patients with lipid metabolism disorders in managing their condition over a 12-month period. The app allowed users to set reminders for medication, track weight, cholesterol levels, and other disease-related variables. | Self-Management. |
| O’Neill et al., 2024 | Asthma | Smartphone App & Web-based portal | Hospital checkout; Patient-provider communication; reminders | Mobile-based application for patients to record ‘selfie’ videos of inhaler use. secure encryption and cloud-based storage for clinician review. password-protected access for healthcare professionals to assess videos and provide feedback. | Self-Management; Personal Health Record System; Information and Education; Collaborative Care. |
| Hietbrink et al., 2023 | Type 2 Diabetes | Smartphone App & Bluetooth-enabled self-measuring device | Symptom tracking; Patient-provider communication | Integrated in the diameter and miguide apps; the solution uses fitbit activity tracker and freestyle libre 2 glucose sensors for monitoring. the app allows users to set goals, track lifestyle, have behavior-based motivational messages, real-time feedback, weekly psychological exercises, and personalized digital coaching. | Self-Management; Digital Therapeutics; Information and Education; Personal Health Record System. |
| Buis et al., 2024 | Hypertension | Smartphone App & Bluetooth-enabled self-measuring device | Activity tracking; Symptom tracking; Medication management; counselling; BP | A mobile app developed by vibrent health, integrated with bluetooth-enabled blood pressure cuff and fitbit zip pedometer. The app supports sodium intake tracking via logging checklist, as well as physical activity tracking, goal setting, educational/motivational messaging, and medication adherence reminders. | Self-Management; Digital Therapeutics; Information and Education; Personal Health Record System. |
| Funes Hernandez et al., 2024 | Hypertension | Smartphone App & Bluetooth-enabled self-measuring device | Symptom tracking; Medication management; Patient-provider communication; BP | Integrated with EHR, includes an evidence-based algorithm for bp control, enables bidirectional data flow, and automates BP monitoring. automated bp tracking, guideline-based medication recommendations, in-basket messaging for clinicians, and workflow integration. | Self-Management; Information and Education; Collaborative Care; Personal Health Record System. |
| Puhong Zhang et al., 2024 | Type 2 Diabetes | Smartphone App & Web-based Portal | Instructional videos; Mental health (self-report); Rate doctor; Patient reports, Goal management | Includes a self-management app for patients and family health promoter, an EHR-integrated clinician decision support system, and data tracking functionalities. Mobile health app for self-monitoring, interactive goal setting, family health promoter-assisted care, and clinician decision support with automatic alerts. | Self-Management; Personal Health Record System; Clinical Decision Support System; Collaborative Care. |
| Chrysohoou et al., 2024 | Chronic Heart Failure | Smartphone App, Bluetooth-enabled self-measuring device & Web-based portal | Symptom tracking; Automated conversation agent; Goal management; Patient-provider communication; Body composition | Mobile health app for heart failure monitoring; provides health data visualization, patient self-reporting tools, and clinician alerts. There are also aspects of tracking clinical, biometric, and biological parameters, interactive patient education, and automated alerts for abnormal readings. Allows for a greater degree of clinical oversight. | Self-Management; Collaborative Care; Digital Therapeutics; Information and Education; Personal Health Record System. |
| Goodman et al., 2024 | Type 2 Diabetes | Smartphone App & Bluetooth-enabled self-measuring device | Instructional videos; Goal management; Patient reports. | Automated phone messaging system; pre-recorded self-care messages; telehealth engagement tracking. The app also contains a diabetes education session via weekly automated calls, tracking of telehealth message engagement, and reinforcement of self-care practices. | Self-Management; Collaborative Care; Information and Education. |
| Wei Zhang et al., 2024 | Type 2 Diabetes Mellitus | Smartphone App & Web-based Portal | Instructional videos; Personal treatment plan; Goal management; Rate doctor | Mobile app for diabetes self-management with a digital dashboard for clinicians and real-time dietary tracking feature. The app contains a Personalized dietary goal setting, with real-time dietary monitoring that can also be interactive with clinician feedback, and general self-management tracking. | Self-Management; Collaborative Care. |
| Babington-Ashaye et al., 2023 | Haemophilia | Smartphone App | AI-driven conversation agent; Instructional videos; | A cross-platform mobile app that is integrated with natural language processing (NLP) for user-friendly interactions, provides self-learning and symptom management. The NLP component acts as an educational chatbot providing real-time responses to haemophilia-related queries, supports disease self-management, and connects users to medical resources. | Self-Management; Information and Education. |
| Rossetto et al., 2023 | Chronic Heart Failure (CHF), Parkinson’s Disease (PD), Chronic Obstructive Pulmonary Disease (COPD) | Tablet App & Bluetooth-enabled self-measuring device | Activity tracking; reminders; Goal management | Tablet-based app delivering rehabilitation activities, integrated with medical devices (activity tracker, bp monitor, balance, pulse oximeter). home-based rehabilitation, remote clinician oversight, activity tracking, and self-management support. | Self-Management; Collaborative Care; Digital Therapeutics. |
| Chun Li et al., 2023 | Rheumatoid Arthritis | Smartphone App, Web-based Portal & Wearable motion sensor | Activity tracking; Medication management; Patient-provider communication | Mobile health app with real-time disease monitoring, patient self-assessment tools, and clinician dashboard. The remote disease activity tracking allows for automated alerts for worsening conditions based on configured parameters. | Self-Management; Information and Education; Collaborative Care; Digital Therapeutics. |
| Genberg et al., 2023 | Asthma | Web-based portal | Activity tracking; Symptom tracking; Patient-provider communication; Messaging | A Web-based portal that is integrated to health village’s "my path" system, which provides asthma education, patient self-monitoring, and clinician messaging. This portal also contains symptom diaries, exacerbation tracking with alerts and inhaler technique guidance. | Self-Management; Collaborative Care; Information and Education. |
| Burka et al., 2023 | Hypertension | Smartphone App & Web-based portal | Warning system; Patient reports; Patient-provider communication; Automated conversation agent | Offline-first mobile app for android for self-management, with a web dashboard for tracking key performance indicators for the clinicians. This includes features such as automated reminders for follow-up visits. The app contains ability for real-time patient registration, tracking of blood pressure, clinician decision support, automated alerts for overdue follow-ups. | Self-Management; Collaborative Care; Clinical Decision Support System. |
| Salim et al., 2023 | Asthma | Smartphone App | Symptom tracking; Medication management; Patient reports. | A mobile app focused on helping patients with asthma. This intervention has underpinnings to Fogg Behaviour gird. The mobile ap acts as an interactive self-management tool with automated reminders, action plan guidance, medication adherence, action plan usage, and behavioral support. | Self-Management; Information and Education; Personal Health Record System. |
| Rusch et al., 2022 | Bipolar Disorder | Smartphone App & Web-based Portal | Symptom tracking; Patient-provider communication | This is a mobile app with mood monitoring functionalities. The app also contains education material that is self-paced, and an action plan tracking. The app contains the ability to track mood trends, coping strategies, and self-assessments with potential provider dashboard integration for further clinical oversight. | Self-Management; Digital Therapeutics; Information and Education; Clinical Decision Support System; Collaborative Care; Personal Health Record System. |
| Barbaric et al., 2022 | Heart Failure | Smartphone App, Web-based Portal & Other sensor devices | Medication management; Patient-provider communication; Symptom tracking; Automated conversation agent; Body composition | This is an Amazon Alexa-hosted application that is linked to the Medly clinical dashboard for real-time patient data input. The patient follows a voice-based interaction to log symptoms, along with other biometric monitoring, automated feedback messages, and integration with Medly smartphone app. | Self-Management; Information and Education; Collaborative Care. |
| Fee et al., 2022 | HIV | Web-based portal, Tracking device & Smartphone App | Appointment management; Messaging. | A mobile and web-based digital case management tool, that allows for real-time communication and document exchange with the healthcare provider. The solution highlights secure document management with other features such as appointment scheduling, messaging, and lab result sharing. | Self-Management; Collaborative Care; Information and Education; Clinical Decision Support System; Collaborative Care. |
| Nabovati et al., 2023 | Type 2 Diabetes | Smartphone App | Symptom tracking; Instructional videos; Patient reports, Appointment management; Medication management | An android only mobile app with self-management functionalities such as logging of nutrition and health data, calculation of insulin doses, educational content, and reminders for medical appointments and glucose monitoring. | Self-Management; Information and Education; Collaborative Care; Personal Health Record System. |
| Tabernero et al., 2022 | Cardiovascular Disease (CVD) | Smartphone App | Messaging; Automated conversation agent | A Whatsapp-based messaging system for psychological interventions, integrated online surveys for follow-ups. This solution contains a daily mindfulness or positive strengthening exercises, interactive feedback, structured self-monitoring. | Self-Management; Collaborative Care. |
| Naranjo-Rojas et al., 2023 | COPD (Chronic Obstructive Pulmonary Disease) | Web-based portal & Smartphone App | Patient reports; Symptom tracking; Patient-provider communication | An android only mobile app and web app that is designed for both patients and healthcare professionals. The mobile contains symptom logging; and the web portal contains a graphical data visualization for clinician decision support. | Self-Management; Collaborative Care; Personal Health Record System. |
| Miki et al., 2024 | Chronic Ischemic Heart Disease (IHD) | Measurement devices, Smartphone App and Web-based Portal | Patient reports; Symptom tracking; Reminders; Activity tracking; Body composition | A Mobile and Web-based digital platform, which is integrated with Fitbit devices and has biometric data tracking capabilities. This allows for the integration of general patient lifestyle and symptom tracking, allowing for lifestyle modification support. Further the app has automated reminders, and generatable physician reports. | Self-Management; Collaborative Care; Personal Health Record System. |
| Holtz et al., 2024 | Type 1 Diabetes | Smartphone App | Symptom tracking; Warning system; Instructional videos; Messaging | A cross-platform mobile app that integrates reminders, messaging, and self-monitoring tools. The app supports tracking type 1 diabetes-related symptoms, monitoring medication adherence, providing interactive educational modules, and enabling family messaging for better disease management. | Self-Management; Collaborative Care; Information and Education; Personal Health Record System. |
| Reid et al., 2023 | Asthma | Smartphone App, Web-based portal & Other sensor devices | Symptom tracking; Instructional videos; Patient reports; Hospital checkout. | A mobile and web-based tool integrated with handheld spirometers and pulse oximeters. It features automated symptom check-ins, spirometry data collection, real-time alerts, physician escalations, and guided asthma action plans for effective respiratory management. | Self-Management; Collaborative Care; Information and Education; Digital Therapeutics; Clinical Decision Support System; Personal Health Record System. |
| Abel et al., 2023 | Hypertension | Smartphone App, Web-based portal & Other sensor devices | Patient reports, Appointment management; Symptom tracking; Medication management; Automated conversation agent; BP | A mobile and web-based platform integrating Omron BP monitors, Fitbit activity trackers, MyFitnessPal, and cloud-based data storage. It enables remote symptom tracking, medication adherence reminders, self-reported food intake monitoring, and automated coaching messages for comprehensive health management. | Personal Health Record System; Collaborative Care; Self-Management; Digital Therapeutics; Information and Education. |
| Hartch et al., 2024 | Various chronic illnesses (hypertension, diabetes, hyperlipidemia, asthma) | Smartphone App | Patient reports.; Medication management; Reminders; Messaging | A mobile app available on iOS and Android, featuring customizable reminders, medication logs, and adherence reports. It supports tracking medication adherence, setting reminders, receiving refill alerts, and accessing an optional peer support feature for enhanced accountability. | Self-Management; Collaborative Care. |
| Xu et al., 2024 | Atrial Fibrillation (AF) | Smartphone App & Web-based Portal | Patient reports; Patient-provider communication; Medication management; Messaging | A mobile app available on iOS and Android, offering educational content, real-time clinician communication, medication tracking, and self-reporting of INR levels. It enables remote anticoagulation monitoring, real-time medication adherence tracking, patient-doctor communication, and educational support for improved treatment management. | Self-Management; Information and Education; Collaborative Care. |
| Xie et al., 2023 | Kidney Transplantation | Smartphone App & Bluetooth-enabled self-measuring device | Instructional videos; Symptom tracking; Medication management; Patient-provider communication; Body composition; Messaging | An Android-based mobile health application integrated with the Huawei Sports Band for biometric tracking. It supports medication adherence tracking, biometric monitoring, social support forums, and clinician follow-ups for comprehensive health management. | Self-Management; Information and Education; Collaborative Care. |
| Xing et al., 2023 | Hypertension | Smartphone App & Web-based Portal | Symptom tracking; Patient-provider communication; Automated conversation agent; counselling; Reminders; BP | A mobile app integrated with community health service platforms and physician dashboards, offering BP self-monitoring, lifestyle change recommendations, virtual consultations, and automated reminders for proactive health management. | Self-Management; Personal Health Record System; Information and Education; Collaborative Care. |
| Díaz-Mohedo et al., 2024 | Chronic Pelvic Pain (CPP) | Smartphone App, Web-based Portal & Bluetooth-enabled self-measuring device | Activity tracking; Patient-provider communication; Symptom tracking; Patient-provider communication | A multi-platform mobile app (iOS, Android, and Web) featuring interactive therapy modules with structured difficulty levels. It offers therapy sessions for left-right discrimination tasks, adaptive difficulty settings, real-time feedback, and session tracking for personalized rehabilitation. | Self-Management; Personal Health Record System; Collaborative Care; Information and Education; Collaborative Care; Digital Therapeutics. |
| Goulding et al., 2023 | Bipolar Disorder | Smartphone App & Web-based Portal | Patient reports; Symptom tracking; Messaging; Medication management; | A mobile app integrated with a clinician dashboard and automated feedback mechanisms, offering mood and medication tracking, wellness planning, self-assessment surveys, and clinician notifications for proactive mental health management. | Self-Management; Information and Education; Collaborative Care; Personal Health Record System. |
| Lalloo et al., 2019 | Chronic Pain | Smartphone App | Goal management; Symptom tracking; Automated conversation agent | A mobile app available for iOS and Android, designed for real-time symptom tracking and feedback. It features symptom tracking, historical data visualization, and self-management education to support proactive health management. | Self-Management; Information and Education; Personal Health Record System. |
| Kim et al., 2020 | Stroke | Smartphone App, Web-based Portal, Bluetooth-enabled self-measuring device | Symptom Tracking; Instructional videos; Patient-provider communication; Symptom tracking; Medication management; BP | A mobile-based mHealth platform integrated with wearable devices and a clinician monitoring dashboard. It enables real-time BP and glucose tracking, symptom self-reporting, educational modules, medication adherence monitoring, and clinician alerts for comprehensive patient care. | Self-Management; Collaborative Care; Information and Education; Personal Health Record System. |

*Self-measurement

web-based portal refers to an online portal through which practitioners can access patient data fed through patient-managed app.

Website refers to interventions that were delivered through a website.

Co-Occurrence Matrix of the digital Intervention strategies used in identified literature

| **Digital Intervention Strategy – Co-Occurrence Matrix** | | | | | | | |
| --- | --- | --- | --- | --- | --- | --- | --- |
|  | **Active Monitoring** | **Clinical Decision Support System** | **Collaborative Care** | **Digital Therapeutics** | **Information and Education** | **Personal Health Record System** | **Self-Management** |
| **Active Monitoring** | 0 | 0 | 0 | 0 | 0 | 1 | 1 |
| **Clinical Decision Support System** | 0 | 0 | 7 | 4 | 5 | 4 | 8 |
| **Collaborative Care** | 0 | 7 | 0 | 12 | 25 | 19 | 47 |
| **Digital Therapeutics** | 0 | 4 | 12 | 0 | 10 | 7 | 15 |
| **Information and Education** | 0 | 5 | 25 | 10 | 0 | 16 | 31 |
| **Personal Health Record System** | 1 | 4 | 19 | 7 | 16 | 0 | 25 |
| **Self-Management** | 1 | 8 | 47 | 15 | 31 | 25 | 0 |
